# Non-local Parabolic and Hyperbolic Models for Cell Polarisation in Heterogeneous Cancer Cell Populations

**DOI:** 10.1007/s11538-018-0477-4

**Published:** 2018-08-22

**Authors:** Vasiliki Bitsouni, Raluca Eftimie

**Affiliations:** Division of Mathematics, University of Dundee, Dundee, DD1 4HN Scotland, UK

**Keywords:** Cancer cells, Non-local hyperbolic model, Parabolic limit, Cell–cell interactions, Alignment, Aggregation patterns, 35R09, 35Q92, 92C15, 92C17, 92-08

## Abstract

Tumours consist of heterogeneous populations of cells. The sub-populations can have different features, including cell motility, proliferation and metastatic potential. The interactions between clonal sub-populations are complex, from stable coexistence to dominant behaviours. The cell–cell interactions, i.e. attraction, repulsion and alignment, processes critical in cancer invasion and metastasis, can be influenced by the mutation of cancer cells. In this study, we develop a mathematical model describing cancer cell invasion and movement for two polarised cancer cell populations with different levels of mutation. We consider a system of non-local hyperbolic equations that incorporate cell–cell interactions in the speed and the turning behaviour of cancer cells, and take a formal parabolic limit to transform this model into a non-local parabolic model. We then investigate the possibility of aggregations to form, and perform numerical simulations for both hyperbolic and parabolic models, comparing the patterns obtained for these models.

## Introduction

Collective cell movement can be observed in many types of cells and plays an important role in many physiological processes, including wound healing, embryonic development and metastasis of cancer cells (Friedl and Wolf 2003; Rørth 2009). Cancer cells’ movement and aggregations are influenced by external factors (e.g. concentration of nutrients), as well as internal factors, such as the social forces among cells (i.e. attraction, repulsion and polarisation). These internal factors lead to self-organised cell aggregation and the formation of a wide variety of patterns, playing a crucial role in cell movement. Experimental studies (Omelchenko et al. 2003; Rørth 2012) have shown that alignment (polarisation) has been reported as the initial cellular response in wound healing and cancer invasion. By alignment, we mean the process where cells turn to adapt their orientation to that of their neighbours, which leads to a polarised group of cells having the same orientation in space and travelling large distances together. In contrast, there are non-polarised groups in which all cells move individually, while the group as a whole can remain stationary or drift slowly (Lutscher 2002; Firtel and Meili 2000). Cells interact with their neighbours and change their shape and direction of movement as a result of this collective movement and a process known as contact inhibition of locomotion (CIL) (Vicente-Manzanares and Sánchez-Madrid 2000), which plays a crucial role in cancer invasion and metastasis. During this process, cells alter their direction of movement when contact other cells in order to avoid collision.

Although the exact mechanism that makes the cells cooperate with each other and migrate collectively in one direction is not fully clear, it has been observed that “leader” cells at the front of outgrowths (e.g. an epithelial cell at the edge of an epithelial sheet that adopts a fibroblast-like morphology extending a wide lamellipodium) are accompanied by many “follower” cells along the sides, both migrating to distant sites (Haga et al. 2005; Omelchenko et al. 2003). As cells move in a collective manner, only the cells in the free edge will produce lamellipodia, while cells inside the group will form smaller protrusions or no protrusion. Although CIL process will lead to a change in the direction of movement of the cells in the edges, the whole group of cells will follow this movement as a result of cell–cell interactions, ending up in the realignment of the cell populations (Mayor and Carmona-Fontaine 2010). Moreover, recent experimental results showed that the polarisation and migration of cells within an epithelial monolayer are coordinated over spatial distances greater than ten cell diameters (Angelini et al. 2010; Petitjean et al. 2010; Das et al. 2015).

In the mathematical literature, there are various models that consider the effect of non-local social interactions on the collective movement of cells and animals. A large number of models for the collective movement of animals consider the interplay between all three social interactions: repulsion, attraction and alignment (Canizo et al. 2010; Cavagna et al. 2010; Gautrais et al. 2012; Huth and Wissel 1992; Kunz and Hemelrijk 2003; Lukeman et al. 2010). Some of these models consider non-local turning rates and constant speeds (see, e.g. Buono and Eftimie 2015; Fetecau 2011). Other models investigate the effect of social interactions also on animals speed (Fetecau and Eftimie 2010; Topaz et al. 2006). In regard to the models for the collective movement of cells, the majority of these models focus on attractive–repulsive interactions (Armstrong et al. 2006; Bitsouni et al. 2017, 2018; Domschke et al. 2014; Painter et al. 2015; Sherratt et al. 2009). Very few non-local models incorporate cell alignment [see, for instance, Mogilner and Edelstein-Keshet (1995)]. Therefore, it is very important to develop non-local models that consider cell polarisation and describe the way that all three social forces affect the velocity and the turning behaviour of cells.

In this paper, we introduce a new model describing the interplay between cell polarisation and cell repulsive–attractive interactions. In contrast to the models mentioned in the previous paragraph, here we consider both non-local speed and turning rates. To this end, we derive a model of nonlinear non-local first-order hyperbolic equations describing the dynamics of polarised early- and late-stage cancer cell populations. In addition to cell movement and cell turning behaviours (which depend on repulsive, attractive and polarising forces), we also consider mutation and proliferation. We investigate numerically the patterns generated by this hyperbolic model—by focusing on the effect of the following parameters: (i) magnitude of repulsive/attractive/polarisation (alignment) interactions; (ii) turning rates; (iii) proliferation rates; and (iv) baseline speed. Since the majority of papers describing collective movement of cells are of parabolic type, in this paper we also take a parabolic limit to investigate the preservation of patterns in this limit.

To keep the model as simple as possible, we focus only on the effect of attraction/repulsion/alignment on cell–cell interactions [while ignoring the interactions between cells and the extracellular matrix (ECM)]. This is consistent with other mathematical approaches in the literature of collective movement of cells [see, e.g. the individual-based models in Arboleda-Estudillo et al. (2010), Chang et al. (2013), Hirashima et al. (2013), Méhes and Vicsek (2014) and Woods et al. (2014)]. We emphasise here that in contrast to these studies that develop discrete models (which are difficult to be investigated analytically), here we present a continuous model where we apply analytical and computational techniques to understand pattern formation in cellular aggregations.

This paper is organised as follows. In Sect. 2, we present a model of non-local nonlinear hyperbolic equations describing the dynamics of two sub-populations of polarised cancer cells, with different levels of mutation. In Sect. 3, we derive the parabolic limit of this hyperbolic model. In Sect. 4, we perform linear stability analysis of both hyperbolic and limiting parabolic models to investigate the ability of these models to form cell aggregation. In Sect. 5, we investigate numerically the spatiotemporal patterns obtained by the hyperbolic model and compare the results with the patterns obtained by the limiting parabolic model. We conclude in Sect. 6 with a discussion of the results.

## A Non-local Hyperbolic Model for Cancer Cell Polarisation

In this section, we introduce a new non-local model that incorporates the tendency of cancer cells to align with other cells that are within a range (alignment range). The model describes the movement of two cancer cell populations, an early- and a late-stage population. Here, we assume that the movement of cancer cells is governed by directed motility in response to cell–cell interactions, choosing to ignore the cell–ECM interactions.

Let $\varOmega \subset \mathbb {R}$ be a bounded interval. Let $I_\mathrm{T}=\left[ 0, \infty \right)$ be the time interval. We denote by $u_1^+\left( t, x\right)$ ($u_1^-\left( t, x\right)$) the density of early-stage cancer cells at $\left( t, x\right)$ that move to the right (left), and, respectively, by $u_2^+\left( t, x\right)$ ($u_2^-\left( t, x\right)$) the density of late-stage cancer cells at $\left( t, x\right)$ that move to the right (left). The total cancer cell population density is given by the relation $u_1=u_1^++u_1^-$ for the early-stage cancer cell population and, respectively, by $u_2=u_2^++u_2^-$ for the late-stage cancer cell population. For compact notation, we define the vector $\underline{u}\left( t, x\right) =\left( u_1\left( t, x\right) , u_2\left( t, x\right) \right) ^\mathrm{T}$. We also define the cell population flows by $\upsilon _i=u_i^+-u_i^-, i=1, 2$, for early-stage ($i=1$) and late-stage ($i=2$) cancer cells. Thus, we derive the following hyperbolic system of conservation laws that describe the evolution of densities of left-moving and right-moving early- and late-stage cancer cells:

1a $$\begin{aligned} \dfrac{\partial u_1^+}{\partial t}+\dfrac{\partial }{\partial x}\left( u_1^+\varGamma ^+\left[ \underline{u}\right] \right)&=-\lambda _{u_1}^+\left[ u_1^+, u_1^-, u_2^+, u_2^-\right] u_1^++\lambda _{u_1}^-\left[ u_1^+, u_1^-, u_2^+, u_2^-\right] u_1^-\nonumber \\&\quad -Mu_1^++r_1\dfrac{u_1}{2}\left( 1-u_1-u_2\right) , \end{aligned}$$

1b $$\begin{aligned} \dfrac{\partial u_1^-}{\partial t}-\dfrac{\partial }{\partial x}\left( u_1^-\varGamma ^-\left[ \underline{u}\right] \right)&=\lambda _{u_1}^+\left[ u_1^+, u_1^-, u_2^+, u_2^-\right] u_1^+-\lambda _{u_1}^-\left[ u_1^+, u_1^-, u_2^+, u_2^-\right] u_1^-\nonumber \\&\quad -Mu_1^-+r_1\dfrac{u_1}{2}\left( 1-u_1-u_2\right) , \end{aligned}$$

1c $$\begin{aligned} \dfrac{\partial u_2^+}{\partial t}+\dfrac{\partial }{\partial x}\left( u_2^+\varGamma ^+\left[ \underline{u}\right] \right)&=-\lambda _{u_2}^+\left[ u_1^+, u_1^-, u_2^+, u_2^-\right] u_2^++\lambda _{u_2}^-\left[ u_1^+, u_1^-, u_2^+, u_2^-\right] u_2^-\nonumber \\&\quad +M\dfrac{u_1}{2}+r_2\dfrac{u_2}{2}\left( 1-u_1-u_2\right) ,\end{aligned}$$

1d $$\begin{aligned} \dfrac{\partial u_2^-}{\partial t}-\dfrac{\partial }{\partial x}\left( u_2^-\varGamma ^-\left[ \underline{u}\right] \right)&=\lambda _{u_2}^+\left[ u_1^+, u_1^-, u_2^+, u_2^-\right] u_2^+-\lambda _{u_2}^-\left[ u_1^+, u_1^-, u_2^+, u_2^-\right] u_2^-\nonumber \\&\quad +M\dfrac{u_1}{2}+r_2\dfrac{u_2}{2}\left( 1-u_1-u_2\right) ,\end{aligned}$$

1e $$\begin{aligned} u_i^\pm \left( 0, x\right)&=u_{i_0}^\pm \left( x\right) \ge 0,\quad i=1, 2, \quad \text{ in }\;\;\varOmega , \end{aligned}$$

where $\varGamma ^\pm \left[ \underline{u}\right]$ are the density-dependent speeds and $\lambda _{u_i}^+$ ($\lambda _{u_i}^-$) are the density-dependent turning rates for the cancer cells initially moving to the right (left) which then turn to the left (right). We denote by *M* the mutation rate of cancer cells and by $r_i,\; i=1, 2$, the proliferation rate of population $u_i$. Note that we consider a non-dimensionalised model, where the cancer cell densities $u_i, i=1, 2$, are non-dimensionalised by the carrying capacity for the cells, $k_u$, leading to logistic growth functions with unit-valued carrying capacity for the cells. A non-dimensionalisation of the model and two tables with the model variables and parameters are presented in “Appendix A”.

The turning rates are functions of the cell–cell interactions, $y^{\pm }\left[ u_1^+, u_1^-, u_2^+, u_2^-\right]$, described as in Eftimie et al. (2007):

2 $$\begin{aligned} \lambda ^{\pm }_{u_i}\left[ u_1^+, u_1^-, u_2^+, u_2^-\right] :=&\,\lambda _i^\mathrm{r}+\lambda _i^\mathrm{b}p\left( y^\pm \left[ u_1^+, u_1^-, u_2^+, u_2^-\right] \right) \nonumber \\ =&\,\lambda _i^\mathrm{r}+\lambda _i^\mathrm{b}\left( 0.5+0.5\tanh \left( y^{\pm }\left[ u_1^+, u_1^-, u_2^+, u_2^-\right] -2\right) \right) , \end{aligned}$$

where the constants $\lambda _i^\mathrm{r}$ and $\lambda _i^\mathrm{b}, i=1, 2$, represent a baseline random turning rate and a biased turning rate, respectively. The dimensionless functionals $y^{\pm }\left[ u_1^+, u_1^-, u_2^+, u_2^-\right]$ (see “Appendix A.1”) of the densities of right-moving, $u_i^+$, and left-moving, $u_i^-$, cancer cells incorporate non-local interactions between the two sub-populations of polarised cells and can be described by the following relation:

3 $$\begin{aligned} y^{\pm }\left[ u_1^+, u_1^-, u_2^+, u_2^-\right]&=y^{\pm }_\mathrm{a}\left[ u_1^+, u_1^-, u_2^+, u_2^-\right] -y^{\pm }_\mathrm{r}\left[ u_1^+, u_1^-, u_2^+, u_2^-\right] \nonumber \\&\quad +y^{\pm }_\mathrm{al}\left[ u_1^+, u_1^-, u_2^+, u_2^-\right] , \end{aligned}$$

where $y^{\pm }_j\left[ u_1^+, u_1^-, u_2^+, u_2^-\right] , j=a, r, al$, denote the attraction, repulsion and alignment functionals, respectively, which influence the likelihood of a cancer cell to turn to the left (+) or to the right (–). We note here that stronger interaction forces lead to higher turning rates. Let us define $R_\mathrm{s}>0$ to be the cells sensing radius, i.e. the maximum range over which cells can detect other surrounding cells, which biologically represent the extent of the cell protrusions (e.g. filopodia) (Armstrong et al. 2006). The attraction and repulsion interactions are described by the following non-local terms (Buono and Eftimie 2015; Colombi et al. 2015, 2017):

4 $$\begin{aligned} y_{\mathrm{a},\mathrm{r}}^{\pm }\left[ \underline{u}\right]&=\dfrac{q_{\mathrm{a},\mathrm{r}}}{R_\mathrm{s}}\int _0^{R_\mathrm{s}} K_{\mathrm{a},\mathrm{r}}\left( s\right) \biggl (u_1\left( t, x\pm s\right) +u_2\left( t, x\pm s\right) -u_1\left( t, x\mp s\right) \nonumber \\&\quad -u_2\left( t, x\mp s\right) \biggr )\mathrm {d}s, \end{aligned}$$

with $q_\mathrm{a}$ and $q_\mathrm{r}$ describing the magnitudes of attractive and repulsive interactions, respectively, and $K_\mathrm{a}\left( x\right)$ and $K_\mathrm{r}\left( x\right)$ describe the spatial ranges over which these interactions take place. We denote by $K(x):=q_{\mathrm{a}}K_{\mathrm{a}}(x)-q_{\mathrm{r}}K_{\mathrm{r}}(x)$ the attraction–repulsion kernel, assuming that it is attractive at medium/long ranges (i.e. at the edges of the cell, and over the neighbouring cells) and repulsive at very short ranges (i.e. over the cell surface).

The non-local alignment term is given by the relation (Buono and Eftimie 2015):

5 $$\begin{aligned} y_\mathrm{al}^{\pm }\left[ u_1^+, u_1^-, u_2^+, u_2^-\right]&=\dfrac{q_\mathrm{al}}{R_\mathrm{s}}\int _0^{R_\mathrm{s}} K_\mathrm{al}\left( s\right) \biggl (u_1^{\mp }\left( t, x\mp s\right) +u_1^{\mp }\left( t, x\pm s\right) \nonumber \\&\quad +u_2^{\mp }\left( t, x\mp s\right) +u_2^{\mp }\left( t, x\pm s\right) -u_1^{\pm }\left( t, x\mp s\right) \nonumber \\&\quad -u_1^{\pm }\left( t, x\pm s\right) -u_2^{\pm }\left( t, x\mp s\right) -u_2^{\pm }\left( t, x\pm s\right) \biggr )\mathrm {d}s, \end{aligned}$$

with $q_\mathrm{al}$ describing the magnitude of alignment and $K_\mathrm{al}\left( x\right)$ describing the spatial range over which alignment takes place.

Let us now focus on the density-dependent speeds $\varGamma ^\pm \left[ \underline{u}\right]$. Here, we choose the non-local speeds of the two-population cancer cells to be described by non-negative, bounded and increasing functionals of the attractive-repulsive cell–cell interactions. Thus $\varGamma ^\pm \left[ \underline{u}\right]$ are given by the following relations

6 $$\begin{aligned} \varGamma ^\pm \left[ \underline{u}\right] =\gamma \left( 1+\tanh \left( y_\mathrm{a}^\pm \left[ \underline{u}\right] -y_\mathrm{r}^\pm \left[ \underline{u}\right] \right) \right) , \end{aligned}$$

where $\gamma$ is a constant baseline speed describing the behaviour of the cancer cell populations in the absence of cell–cell interactions (see Fetecau and Eftimie 2010). We denote by $g\left[ \underline{u}\right] :=\tanh \left( y^+\left[ \underline{u}\right] \right)$. Since function $\tanh \left( \cdot \right)$ is an odd function, then for $q_\mathrm{al}=0$ relation (6) becomes:

7 $$\begin{aligned} \varGamma ^\pm \left[ \underline{u}\right] =\gamma \left( 1\pm g\left[ \underline{u}\right] \right) . \end{aligned}$$

For the non-local terms, we choose translated Gaussian kernels

8 $$\begin{aligned} K_j\left( x\right) =\dfrac{1}{\sqrt{2\pi m^2_{j}}}\hbox {e}^{-\frac{\left( x-s_{j}\right) ^2}{2m^2_{j}}},\;\;\; j=a, r, al, \end{aligned}$$

with $s_j$ representing half the length of the interaction ranges and $m_j=s_j/8$ representing the widths of the interaction kernels [the constants $m_j, j=a,r, al$, are chosen such that the support of more than $98\%$ of the mass of the kernels is inside the interval $\left[ 0, \infty \right)$ (Eftimie et al. 2007)]. Note that other studies used discontinuous Morse-type repulsion–attraction kernels (Fetecau and Eftimie 2010), which have a more realistic shape (with highest repulsion at $x=0$), but which can cause density blow-up [a different class of repulsion–attraction kernels in higher dimensions, which are also discontinuous at the origin where they have the highest density, but which are always positive (in contrast to the more classical Morse kernels that can be positive and/or negative depending on parameter values), was recently discussed by Carrillo et al. (2016)]. To avoid this type of unrealistic aggregation behaviour, we have chosen translated Gaussian kernels (8).

We study the hyperbolic model (1) on a finite domain of length *L*, that is, $x\in \left[ 0, L\right]$, with wrap-around boundary conditions for the non-local social interactions. Thus, we have a problem with a discrete spectrum, where for *L* large we can approximate the process of pattern formation on an unbounded domain. To complete the model, we have to impose boundary conditions. Note that since system (1) is hyperbolic, we have to follow the characteristics of the system when imposing these boundary conditions. For this reason, $u^+_i, \; i=1, 2$, are prescribed only at $x=0$, while $u^-_i, \; i=1, 2$ are prescribed only at $x=L$. For this model, we choose periodic boundary conditions, where the cancer cells move on a circular domain, leaving the domain at one end and entering it again at the other end. The boundary conditions are described by:

9 $$\begin{aligned} u_i^+\left( t, 0\right) =u_i^+\left( t, L\right) \;\text{ and }\; u_i^-\left( t, L\right) =u_i^-\left( t, 0\right) ,\; i=1, 2. \end{aligned}$$

## Parabolic Limit for Non-local Interactions

In this section, we take a formal parabolic limit to investigate the connection between the hyperbolic model (1) and other published non-local parabolic models for collective cell dynamics, which have density-dependent speed (see, e.g. Domschke et al. 2014; Painter et al. 2015 and the references therein). To study the parabolic limit of our hyperbolic model, we assume that there is no alignment, i.e. $q_\mathrm{al}=0$. The main reason for choosing to ignore alignment is that we aim to obtain closed-form parabolic equations for the total densities of cancer cells ($u_{1,2}$), and the alignment terms (for $q_\mathrm{al}\ne 0$) incorporate left- and right-moving cancer cells (which would lead to terms depending on the flow $v_{1,2}$).

Recent experimental results on cancer cell motility have shown that there are differences in terms of speed and directional persistence between 2D and 3D cell migrations (Wu et al. 2014). Moreover, while cells have been observed to move persistently in 2D at short time scales (i.e. their behaviour corresponding to hyperbolic dynamics), they displayed correlated random walk at long time scales (i.e. their behaviour corresponding to parabolic dynamics). We can explain the experimental behaviour observed on short and long time scales by taking the parabolic limit of the hyperbolic model (1). As discussed in Hillen and Painter (2013), there are two approaches for this parabolic limit: (i) an appropriate scaling of space and time and (ii) large turning rates and large speeds. Since the two approaches are equivalent (Hillen and Painter 2013), throughout this study we chose to focus on the scaling of the turning rates and speeds.

To transform model (1) into a parabolic model, we follow the classical approach in (Kac 1974; Hillen and Stevens 2000) and differentiate with respect to *t* and *x* the sum and difference of Eqs. (1a)–(1b) and also Eqs. (1c)–(1d). After eliminating the equations for the cell fluxes ($v_{1}=u_{1}^{+}-u_{1}^{-}$ and $v_{2}=u_{2}^{+}-u_{2}^{-}$), we are left with two equations for the total densities of cancer cells ($u_{1,2}=u_{1,2}^{+}+u_{1,2}^{-}$)—see Eqs. (51) and (52) in “Appendix B” (as well as the details of the calculations shown in “Appendix B”).

Next, we rescale the turning rates and speeds, by assuming that the cancer cells move very fast and change direction even faster [with respect to other normal cells in the tissue; see also the experimental study in Tzvetkova-Chevolleau et al. (2008)]. Moreover, since it makes sense to assume that high speeds and high turning rates lead also to a reduced sensitivity to the environment (and implicitly to other neighbouring cells), we consider also a scaling of the density-dependent components of the speed [i.e. the term *g*[*u*] that appears in $\varGamma ^{\pm }[u]=\gamma (1\pm g[u])$] and of the turning rates (i.e. the term $p(y^{\pm }[u])$ that appears in $\lambda ^{\pm }_{u_{1,2}}$) [see also the approach in Buono and Eftimie (2015)].

With these assumptions, we introduce a small dimensionless parameter $\epsilon >0$ and use it for the following rescaling

(i) $$\lambda ^\mathrm{r}_i=\dfrac{\bar{\lambda }_i^{\mathrm{r}}}{\epsilon ^2},\;\;\; \lambda ^\mathrm{b}_i=\dfrac{\bar{\lambda }_i^{\mathrm{b}}}{\epsilon ^2},\; i=1, 2$$,
(ii) $$\gamma =\dfrac{\bar{\gamma }}{\epsilon }$$,
(iii) $$g\left[ \underline{u}\right] =\epsilon \bar{g}\left[ \underline{u}\right] $$,
(iv) $$p\left( y^\pm \left[ \underline{u}\right] \right) =\epsilon \bar{p}\left( y^\pm \left[ \underline{u}\right] \right) $$.

We denote by $f_i\left[ \underline{u}\right] =\lambda _{u_i}^-\left[ u_1^+, u_1^-, u_2^+, u_2^-\right] -\lambda _{u_i}^+\left[ u_1^+, u_1^-, u_2^+, u_2^-\right]$ and $h_i\left[ \underline{u}\right] =\lambda _{u_i}^-\left[ u_1^+, u_1^-, u_2^+, u_2^-\right] +\lambda _{u_i}^+\left[ u_1^+, u_1^-, u_2^+, u_2^-\right]$. This reduction in the sensitivity in the environment leads to the following rescaling

10 $$\begin{aligned}&\bullet f_i\left[ \underline{u}\right] =\dfrac{\bar{\lambda }_i^{\mathrm{b}} \bar{f}\left[ \underline{u}\right] }{\epsilon }, \;\;\text{ with }\;\; \bar{f}\left[ \underline{u}\right] =\bar{p}\bigl (y^-\left[ \underline{u}\right] \bigr )-\bar{p}\bigl (y^+\left[ \underline{u}\right] \bigr ),\; i=1, 2, \end{aligned}$$

11 $$\begin{aligned}&\bullet h_i\left[ \underline{u}\right] =\dfrac{2\bar{\lambda }_i^\mathrm{r} +2\bar{\lambda }_i^{\mathrm{b}}\epsilon \bar{h}\left[ \underline{u}\right] }{\epsilon ^2}, \;\;\text{ with }\;\; \bar{h}\left[ \underline{u}\right] =\bar{p}\bigl (y^-\left[ \underline{u}\right] \bigr )+\bar{p}\bigl (y^+\left[ \underline{u}\right] \bigr ),\; i=1, 2. \end{aligned}$$

We then substitute these rescaled parameters and functions into a reduced system for the total cell densities $u_{1,2}=u_{1,2}^{+}+u_{1,2}^{-}$, which was obtained from model (1); see Eqs. (51)–(52) in “Appendix B”. This approach has been previously described in detail in Hillen and Levine (2003) and Hillen and Stevens (2000). As we then take the limit $\epsilon \rightarrow 0$ in this simplified system, we obtain the following parabolic equations

12a $$\begin{aligned}&\dfrac{\partial u_1}{\partial t}=D_{u_1}\dfrac{\partial ^2 u_1}{\partial x^2}-\dfrac{\gamma \lambda _1^\mathrm{b}}{2\lambda _1^\mathrm{r}}\dfrac{\partial }{\partial x}\left( u_1f\left[ \underline{u}\right] \right) -\gamma \dfrac{\partial }{\partial x}\left( u_1g\left[ \underline{u}\right] \right) -Mu_1+R_1\left( \underline{u}\right) , \end{aligned}$$

12b $$\begin{aligned}&\dfrac{\partial u_2}{\partial t}=D_{u_2}\dfrac{\partial ^2 u_2}{\partial x^2}-\dfrac{\gamma \lambda _2^\mathrm{b}}{2\lambda _2^\mathrm{r}}\dfrac{\partial }{\partial x}\left( u_2f\left[ \underline{u}\right] \right) -\gamma \dfrac{\partial }{\partial x}\left( u_2g\left[ \underline{u}\right] \right) +Mu_1+R_2\left( \underline{u}\right) , \end{aligned}$$

where $D_{u_i}=\dfrac{\left( \gamma \right) ^2}{2\lambda ^\mathrm{r}_i},\; i=1, 2$, are the diffusion coefficients, and $R_i\left( \underline{u}\right) =r_iu_i\left( 1-u_1-u_2\right)$, the growth functions. Here, the initial conditions are given by the functions $u_i\left( 0, x\right) =u_{i_0}\left( x\right) \ge 0, \; i=1, 2$.

To fully define the parabolic model (12), we need to impose boundary conditions. To be consistent with the hyperbolic model (1), we impose again periodic boundary conditions on a finite domain of length *L*:

13 $$\begin{aligned} u_1\left( t, 0\right) =u_1\left( t, L\right) \;\text{ and }\; u_2\left( t, 0\right) =u_2\left( t, L\right) . \end{aligned}$$

We note that, since we assumed $q_\mathrm{al}=0$, the non-local terms $f\left[ \underline{u}\right]$ and $g\left[ \underline{u}\right]$ now depend only on the repulsive and attractive interactions.

## Linear Stability Analysis

In this section, we investigate the possibility of pattern formation for models (1) and (12) via linear stability analysis. To this end, we focus on model parameters, including the magnitudes of social forces (i.e. attraction, repulsion, alignment) between cancer cells, and their role on pattern formation.

### Linear Stability Analysis of the Hyperbolic Model

We start with the linear stability analysis of the hyperbolic model (1). First, we look for the spatially homogeneous steady states $u^{\pm , *}_i, i=1, 2$, assuming that cancer cells are spread evenly over the domain. We denote the constant total density of the populations by $u^*_i, i=1, 2$. From the right-hand side of Eqs. (1a)–(1d), we have the following system:

14a $$\begin{aligned} -Mu_1^*+r_1u_1^*\left( 1-u_1^*-u_2^*\right) =0, \end{aligned}$$

14b $$\begin{aligned} Mu_1^*+r_2u_2^*\left( 1-u_1^*-u_2^*\right) =0, \end{aligned}$$

which has the solutions $\left( u_1^*, u_2^*\right) =\left( 0, 0\right)$ and $\left( u_1^*, u_2^*\right) =\left( 0, 1\right)$. Note that, for biological realism, we consider only non-negative solutions. If we consider the states where both cell populations are evenly spread in both directions over the domain, then these states $\left( u_1^{+, *}, u_1^{-, *}, u_2^{+, *}, u_2^{-, *}\right)$ are given by

15 $$\begin{aligned} \left( 0, 0, 0, 0\right) \;\; \text{ and }\;\; \left( 0, 0, 0.5, 0.5\right) . \end{aligned}$$

If we consider populations that are evenly spread over the domain, but where more individuals are facing one direction compared to the other direction (i.e. $u_{2}^{+,*}\ne u_{2}^{-,*}$), then the steady states are given by

16 $$\begin{aligned} \left( 0, 0, 0, 0\right) \;\; \text{ and }\;\; \left( 0, 0, u_2^{+, *}, 1-u_2^{+, *}\right) , \end{aligned}$$

for $$0\le u_2^{+, *}\le 1$$.

Now that we know the steady states, we proceed with the study of the local stability of these solutions under small perturbations caused by spatially non-homogeneous terms. We let $u_1^\pm =u_1^{\pm , *}+A_{u_1}^\pm \hbox {e}^{ikx+\lambda t}$ and $u_2^\pm =u_2^{\pm , *}+A_{u_2}^\pm \hbox {e}^{ikx+\lambda t}$ with $|A_{u_1}^\pm |, |A_{u_2}^\pm |\ll 1$, where *k* and $\lambda$ are the wave number and frequency, respectively. Due to the finite domain (with wrap-around boundary conditions), we have that the wave number, *k*, takes only discrete values $k_j=2pj/L,\; j=1, 2, 3, \dots$. Let $\hat{K}^\pm _j, j=a, r, al$, be the Fourier transform of the interaction kernel $K_j$, given by the following relation

17 $$\begin{aligned} \hat{K}_j^\pm \left( k\right) =\int _{-\infty }^{\infty } K_j\left( s\right) \hbox {e}^{\pm iks_j}\mathrm {d}s,\;\; j=a, r, al. \end{aligned}$$

We denote by $\hat{K^\mathrm{s}}\left( k\right) =\hat{K}^+\left( k\right) -\hat{K}^-\left( k\right) =q_\mathrm{a}\hat{K^\mathrm{s}}_\mathrm{a}\left( k\right) -q_\mathrm{r}\hat{K^\mathrm{s}}_\mathrm{r}\left( k\right)$ the Fourier sine transform of kernel *K*, and, respectively, by $\hat{K^\mathrm{c}}_\mathrm{al}\left( k\right) =\hat{K}^+_\mathrm{al}\left( k\right) +\hat{K}^-_\mathrm{al}\left( k\right)$ the Fourier cosine transform of kernel $K_\mathrm{al}$. Throughout this study, we will consider translated Gaussian kernels given by relation (8). Then, the Fourier transform of these kernels is given by

18 $$\begin{aligned} \hat{K}_j^\pm \left( k\right) =\exp \left( \pm iks_j-k^2m^2_j/2\right) ,\;\; j=a, r, al, \end{aligned}$$

and the Fourier sine and cosine transforms are given by

19 $$\begin{aligned} \hat{K}_j^\mathrm{s}\left( k\right) =\exp \left( -k^2m^2_j/2\right) \sin \left( ks_j\right) ,\;\; \text{ and }\;\; \hat{K}_j^\mathrm{c}\left( k\right) =\exp \left( -k^2m^2_j/2\right) \cos \left( ks_j\right) .\nonumber \\ \end{aligned}$$

To simplify the results of this section (using the fact that $u_1^{+, *}=u_1^{-, *}=0$), we set the following parameter values:

20 $$\begin{aligned} L_i^\pm&=\lambda _i^\mathrm{r}+0.5\lambda _i^\mathrm{b}+0.5\lambda _i^\mathrm{b}\tanh \left( \pm Q^*-2\right) ,\;i=1, 2,\nonumber \\ Q^*&=\dfrac{2q_\mathrm{al}}{R_\mathrm{s}}\left( u_2^{-, *}-u_2^{+, *}\right) ,\nonumber \\ f\left( u\right)&=0.5\tanh \left( u\right) ,\nonumber \\ f'\left( u\right)&=0.5-2f^2\left( u\right) ,\nonumber \\ B&=u^{+, *}_2f'\left( Q^*-2\right) +u^{-, *}_2f'\left( -Q^*-2\right) ,\nonumber \\ Y\left( k\right)&=-\dfrac{2k\gamma }{R_\mathrm{s}}\hat{K^\mathrm{s}}\left( k\right) ,\nonumber \\ W^\pm \left( k\right)&=\dfrac{B\lambda _2^\mathrm{b}}{R_\mathrm{s}}\left[ i\hat{K^\mathrm{s}}\left( k\right) \mp q_\mathrm{al}\hat{K^\mathrm{c}}_\mathrm{al}\left( k\right) \right] . \end{aligned}$$

Substituting now the expressions $u_j^\pm =u_j^{\pm , *}+A_{u_j}^\pm \hbox {e}^{ikx+\lambda t}, \; j=1, 2$, into the system (1) and using the above relations, we obtain the following dispersion relations:

- For the steady state $\left( 0, 0, 0, 0\right)$, we have:
  21 $$\begin{aligned} \left( \lambda ^2+\lambda D_1\left( k\right) +E_1\left( k\right) \right) \cdot \left( \lambda ^2+\lambda D_2\left( k\right) +E_2\left( k\right) \right) =0 \end{aligned}$$
  with
  22 $$\begin{aligned} D_1\left( k\right)&=L_1^++L_1^-+2M-r_1, \end{aligned}$$
  23 $$\begin{aligned} E_1\left( k\right)&=k^2\gamma ^2-\left( L_1^+-L_1^-\right) ik\gamma \nonumber \\&\quad +\left( L_1^++L_1^-+M-r_1\right) M-\left( L_1^++L_1^-\right) r_1, \end{aligned}$$
  and
  24 $$\begin{aligned} D_2\left( k\right)&=L_2^++L_2^--r_2, \end{aligned}$$
  25 $$\begin{aligned} E_2\left( k\right)&=k^2\gamma ^2-\left( L_2^+-L_2^-\right) ik\gamma -\left( L_2^++L_2^-\right) r_2. \end{aligned}$$
- For the steady state $\left( 0, 0, u_2^{+, *}, 1-u_2^{+, *}\right)$, we have:
  26 $$\begin{aligned} \left( \lambda ^2+\lambda D_3\left( k\right) +E_3\left( k\right) \right) \cdot \left( \lambda ^2+\lambda D_4\left( k\right) +E_4\left( k\right) \right) =0 \end{aligned}$$
  with
  27 $$\begin{aligned} D_3\left( k\right)&=L_1^++L_1^-+2M, \end{aligned}$$
  28 $$\begin{aligned} E_3\left( k\right)&=k^2\gamma ^2-\left( L_1^+-L_1^-\right) ik\gamma +\left( L_1^++L_1^-+M\right) M, \end{aligned}$$
  and
  29 $$\begin{aligned} D_4\left( k\right)&=L_2^++L_2^-+r_2+W^+\left( k\right) -W^-\left( k\right) +Y\left( k\right) , \end{aligned}$$
  30 $$\begin{aligned} E_4\left( k\right)&=k^2\gamma ^2-\left( L_2^+-L_2^-+W^+\left( k\right) +W^-\left( k\right) +\left( 2u_2^{+, *}-1\right) Y\left( k\right) \right) ik\gamma \nonumber \\&\quad +\left( L_2^++L_2^-+W^+\left( k\right) -W^-\left( k\right) \right) \left( Y\left( k\right) +r_2\right) . \end{aligned}$$

**Fig. 1:**
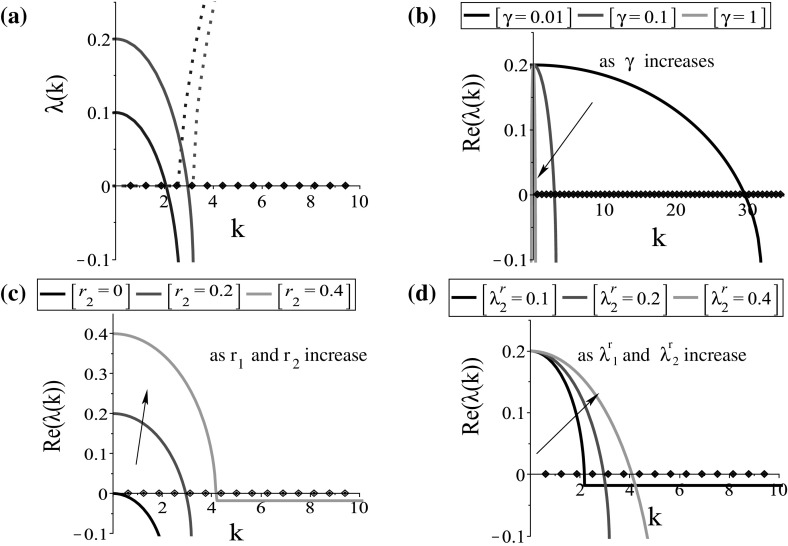
The dispersion relation (21) for the steady state $\left( 0, 0, 0, 0\right)$. **a** Plot of the larger eigenvalues $\lambda _l\left( k\right) =\left( -D_l\left( k\right) +\sqrt{D_l^2\left( k\right) -4E_l\left( k\right) }\right) /2,\;l=1,2$, obtained by dispersion relations (21) for $D_1,\; E_1$ (blue) and $D_2,\; E_2$ (red); **b** the effect of $\gamma$ on the graph of $Re\left( \lambda _2\left( k\right) \right)$; **c** the effect of $r_2$ on the graph of $Re\left( \lambda _2\left( k\right) \right)$; **d** the effect of $\lambda ^\mathrm{r}_2$ on the graph of $Re\left( \lambda _2\left( k\right) \right)$. The continuous curves represent the $Re(\lambda (k))$, while the dotted curves represent the $Im(\lambda (k))$. The model parameters are given in Table 2. The diamonds on the *x*-axis represent the discrete wave numbers $k_j=2\pi j/L, j=1, 2, \dots$ (Color figure online)

**Fig. 2:**
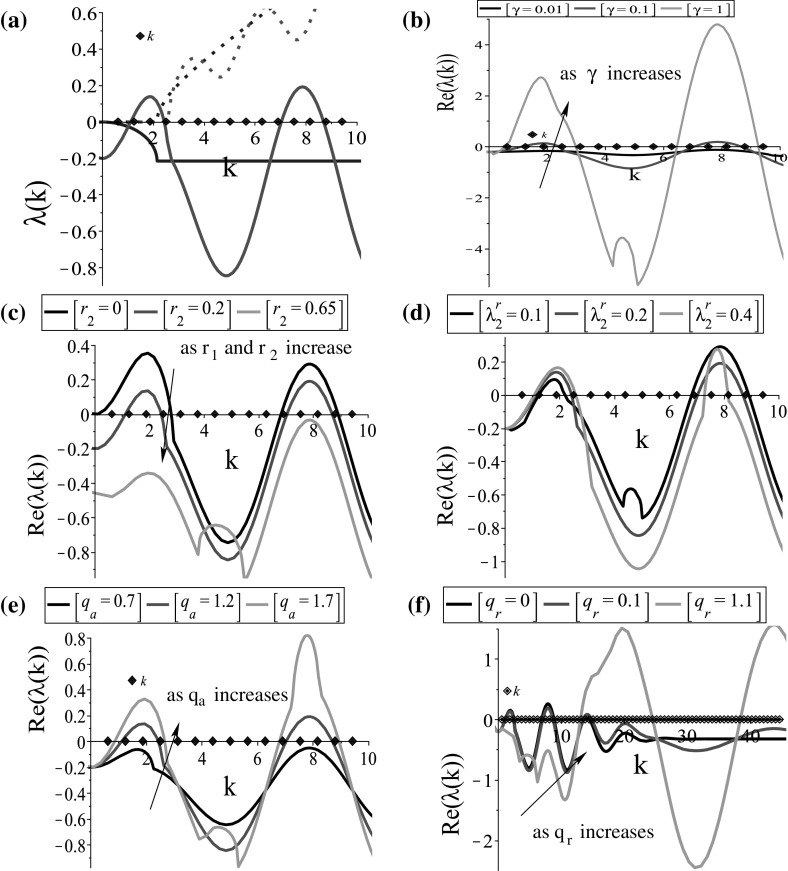
The dispersion relation (26) for the steady state $\left( 0, 0, 0.5, 0.5\right)$. **a** Plot of the larger eigenvalues $\lambda _l\left( k\right) =\left( -D_l\left( k\right) +\sqrt{D_l^2\left( k\right) -4E_l\left( k\right) }\right) /2,\;l=3,4$, obtained by dispersion relations (26) for $D_3,\; E_3$ (blue) and $D_4,\; E_4$ (red); **b** the effect of $\gamma$ on the graph of $Re\left( \lambda _4\left( k\right) \right)$; **c** the effect of $r_2$ on the graph of $Re\left( \lambda _4\left( k\right) \right)$; **d** the effect of $\lambda ^\mathrm{r}_2$ on the graph of $Re\left( \lambda _4\left( k\right) \right)$; **e** the effect of $q_\mathrm{a}$ on the graph of $Re\left( \lambda _4\left( k\right) \right)$; **f** the effect of $q_\mathrm{r}$ on the graph of $Re\left( \lambda _4\left( k\right) \right)$. The continuous curves represent the $Re(\lambda (k))$, while the dotted curves represent the $Im(\lambda (k))$. The model parameters are given in Table 2. The diamonds on the *x*-axis represent the discrete wave numbers $k_j=2\pi j/L, j=1, 2, \dots$ (Color figure online)

Equations (21) and (26) show that the steady states are unstable, i.e. $Re\left( \lambda \left( k\right) \right) >0$, when $D_l\left( k\right) <0$ or $E_l\left( k\right) <0, \; l=1,\dots , 4$. Examples of such dispersion relations are shown in Figs. 1a and 2a. There is a range of *k*-values for which $Re\left( \lambda \left( k\right) \right)$ is positive, and thus, aggregation can arise from spatial perturbations of the steady states $\left( 0, 0, 0, 0\right)$ (see Fig. 1a) and $\left( 0, 0, 0.5, 0.5\right)$ (see Fig. 2a). Note that similar results (not shown here) are obtained for any steady state $\left( 0, 0, u_2^{+, *}, 1-u_2^{+, *}\right)$, with $0\le u_2^{+, *}\le ~1$.

*The effect of the parameters on the stability of the steady states* We now use the dispersion relations (21) and (26) to study the effect of the key parameters on pattern formation. We investigate the stability of the spatially homogeneous steady states $\left( 0, 0, 0, 0\right)$ and $\left( 0, 0, 0.5, 0.5\right)$ by increasing (or decreasing) the parameters connected to the dispersion relations. Precisely, we show the effect of the parameters on the graph of the eigenvalue with the maximum real part, i.e. $Re\left( \lambda _2\left( k\right) \right)$ and $Re\left( \lambda _4\left( k\right) \right)$ of the dispersion relations (21) and (26), respectively. However, we note that the effect of the parameters (e.g. $\lambda ^\mathrm{r}_1$ and $r_1$, and the rest parameters) is similar on the graphs of $Re\left( \lambda _1\left( k\right) \right)$ and $Re\left( \lambda _3\left( k\right) \right)$.

For the tumour-free steady state $\left( 0, 0, 0, 0\right)$, we can see from the dispersion relation (21) that its stability does not depend on the magnitudes of cell interactions, although these magnitudes are crucial in the nonlinear interactions that control cell aggregation patterns. In Fig. 1b, c, d, we see that the stability of this steady state depends mainly on the baseline speed, the proliferation rates and the baseline random turning rates, with the latter not having a significant impact on the instability of the steady state. Precisely, for zero proliferation rates ($r_{1}=r_{2}=0$) the steady state is stable (see Fig. 1c), while an increase in them or in the baseline random turning rates ($\lambda _{1,2}^\mathrm{r}$) results in a shift to the right of the wave number that will emerge. Note also that an increase in the baseline speed ($\gamma$) has an inverse result in the dispersion relation, with a shift to the left of the wave number that will emerge.

For the steady state $\left( 0, 0, 0.5, 0.5\right)$, there are more parameters that affect the stability properties and as it can be seen by relations (29) and (30) the stability of this steady state depends on the magnitudes of cell interactions as well. We can see in Fig. 2b, c that the baseline speed and the proliferation rates have an opposite effect on the stability changes of $\left( 0, 0, 0.5, 0.5\right)$, compared to that on the cancer-free steady state. In Fig. 2e, f we see that a decrease in the magnitude of attraction leads to the change in the stability of this steady state, while an increase in the magnitude of repulsion leads to the shift of the critical wave numbers to the right. Note that although the magnitude of alignment does not seem to affect the stability wave number, it is crucial though in the pattern formation, as we will see in the following section.

#### Remark 1

We should mention here that the effect of the mutation rate, *M*, on the dispersion relation is not significant. Precisely, as *M* appears only on the functions $D_1, E_1$ and $D_3, E_3$, any changes in the values of *M* will not lead to stability change, but only to a reduction on the eigenvalues $\lambda _1$ and $\lambda _3$ (up to below zero) as *M* increases. Note that we always refer to the greater eigenvalues of Eqs. (21) and (26), given by the relation $\lambda _l\left( k\right) =\left( -D_l\left( k\right) +\sqrt{D_l^2\left( k\right) -4E_l\left( k\right) }\right) /2, l=1,\dots ,4$.

**Fig. 3:**
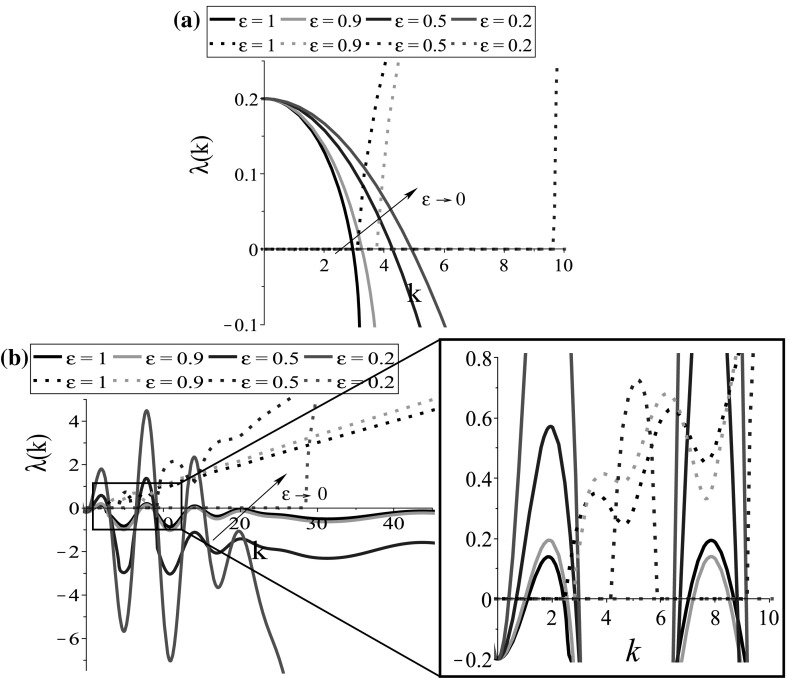
Plot of the eigenvalue with the maximum real part, after parabolic scaling, of **a** relation (21) for the s.s. $\left( 0, 0, 0, 0\right)$ for $q_\mathrm{al}=0$; **b** relation (26) for the s.s. $\left( 0, 0, 0.5, 0.5\right)$ for $q_\mathrm{al}=0$. The continuous curves represent the $Re(\lambda (k))$, while the dotted curves represent the $Im(\lambda (k))$. The rest of the model parameters are given in Table 2. For graphical purposes, the discrete wave numbers has been omitted (Color figure online)

To investigate the effect of the scaling parameter $\epsilon$ on the dispersion relation, in Fig. 3a, b we show the stability of the steady states $\left( 0, 0, 0, 0\right)$ and $\left( 0, 0, 0.5, 0.5\right)$, respectively, after applying the rescaling given in Sect. 3 and taking $q_\mathrm{al}=0$. Although the wave numbers $k_j=2\pi j/L, \;j=1, 2, \dots$ are discrete (represented by diamond-shaped points in *x*-axis of Figs. 1 and 2), here they are plotted as a continuous axis to show clearly the effect of $\epsilon$ on the imaginary part of the dispersion relation described by dotted curves. We see in Fig. 3b that for $\epsilon =1$ there are wave numbers for which we can have $Re\left( \lambda \left( k_j\right) \right) >0$ and $Im\left( \lambda \left( k_j\right) \right) >0$, giving rise to travelling patterns. As $\epsilon$ decreases (e.g. $\epsilon =0.5$), we note that $Im\left( \lambda \left( k_j\right) \right) =0$ at some wave numbers where $Re\left( \lambda \left( k_j\right) \right) >0$, and thus stationary pulses are expected to be obtained. As $\epsilon \rightarrow 0$, the imaginary part of the eigenvalues will be always zero, and numerically we expect to observe stationary patterns.

### Linear Stability Analysis of the Parabolic Model

Next, we investigate the conditions under which aggregations can arise for the limiting parabolic model (12). We first calculate the spatially homogeneous steady states of the parabolic model. We see from Eqs. (12a)–(12b) that the ODE model associated with system (12) is described by system (14), which has the solutions $\left( u_1^*, u_2^*\right) =\left( 0, 0\right)$ and $\left( 0, 1\right)$ (considering again only non-negative solutions).

Proceeding with the linear stability analysis of the spatial system (12), we apply small spatial perturbations to the homogeneous steady states: $u_1=u_1^*+A_{u_1}\hbox {e}^{ikx+\lambda t}$ and $u_2=u_2^*+A_{u_2}\hbox {e}^{ikx+\lambda t}$ with $|A_{u_1}|, |A_{u_2}|\ll 1$. Substituting these terms into system (12), using the parameter values (20) and replacing $u_1^*=0$, yields the following dispersion relation:

31 $$\begin{aligned}&\left[ -\ k^2D_{u_1}-M+r_1\left( 1-u_2^*\right) -\lambda \right] \cdot \Bigg [-k^2D_{u_2}+Y\left( k\right) \Bigg (\dfrac{\lambda _2^\mathrm{b}f'\left( -2\right) }{2\lambda _2^\mathrm{r}}-1\Bigg )u^*_2\nonumber \\&\quad +r_2\left( 1-2u_2^*\right) -\lambda \Bigg ]=0. \end{aligned}$$

**Fig. 4:**
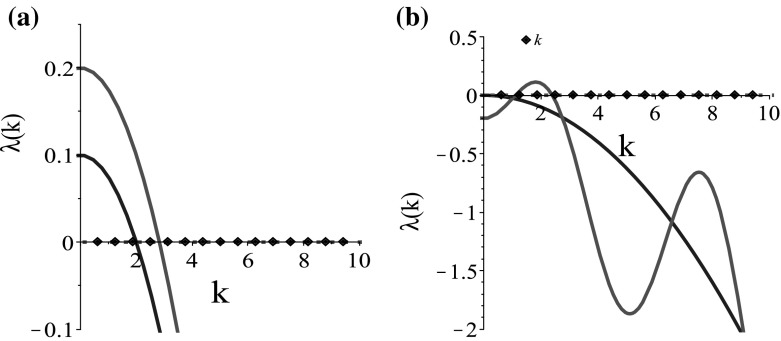
Plot of the eigenvalues obtained by dispersion relation (31). **a** $\lambda _1\left( k\right) =-k^2D_{u_1}-M+r_1$ (blue) and $\lambda _2\left( k\right) =-k^2D_{u_2}+r_2$ (red) for the steady state $\left( 0, 0\right)$; **b** $\lambda _1\left( k\right) =-k^2D_{u_1}-M$ (blue) and $\lambda _2\left( k\right) =-k^2D_{u_2} +Y\left( k\right) \left( \lambda _2^\mathrm{b}f'\left( -2\right) /(2\lambda _2^\mathrm{r})-1\right) -r_2$ (red), for the steady state $\left( 0, 1\right)$. The model parameters are given in Table 2. The continuous curves represent the $Re\left( \lambda (k)\right)$, as the imaginary part of the eigenvalues is zero (represented by dotted lines) in the case of the parabolic model [see relations (31)–(33)]. The diamonds on the *x*-axis represent the discrete wave numbers $k_j=2\pi j/L, j=1, 2, \dots$ (Color figure online)

**Fig. 5:**
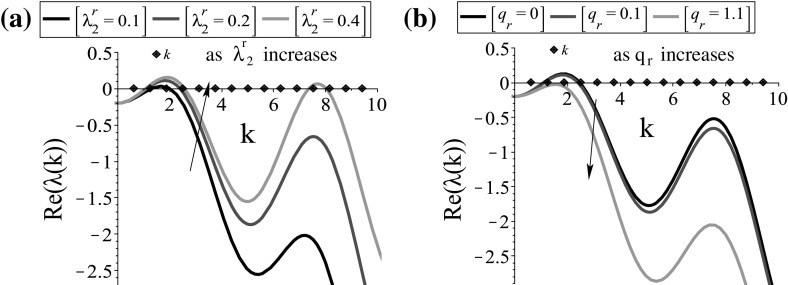
The dispersion relation (31) for the steady state $\left( 0, 1\right)$. **a** The effect of $\lambda _2^\mathrm{r}$ on the graph of $Re\left( \lambda _2\left( k\right) \right)$; **b** the effect of $q_\mathrm{r}$ on the graph of $Re\left( \lambda _2\left( k\right) \right)$; the rest of the model parameters are given in Table 2. The diamonds on the *x*-axis represent the discrete wave numbers $k_j=2\pi j/L, j=1, 2, \dots$ (Color figure online)

Therefore, for the steady state $\left( 0, 0\right)$ we have the solutions:

32 $$\begin{aligned} \lambda _1=-k^2D_{u_1}-M+r_1\;\; \text{ and }\;\; \lambda _2=-k^2D_{u_2}+r_2, \end{aligned}$$

and for the steady state $\left( 0, 1\right)$, the solutions:

33 $$\begin{aligned} \lambda _1=-k^2D_{u_1}-M<0 \;\; \text{ and }\;\; \lambda _2=-k^2D_{u_2} +Y\left( k\right) \left( \dfrac{\lambda _2^\mathrm{b}f'\left( -2\right) }{2\lambda _2^\mathrm{r}}-1\right) -r_2.\nonumber \\ \end{aligned}$$

As in the case of the hyperbolic model, we see in Fig. 4 that there is a range of *k*-values for which $Re\left( \lambda \left( k\right) \right) >0$, and thus, aggregation can arise from spatial perturbations of the steady states $\left( 0, 0\right)$ and $\left( 0, 1\right)$ of the parabolic model. However, taking into account that we have $q_\mathrm{al}=0$ and all eigenvalues have zero imaginary part, we expect that the numerical simulations will show stationary patterns.

Figures 1a and 4a, as well as Figs. 2a and 4b, are similar, and the effect of the key parameters on the dispersion relation is the same. In Fig. 5, we only include the parameters which seem to have a slightly different effect on the dispersion relation for the steady state $\left( 0,1\right)$ of the parabolic model, compared to the one of the hyperbolic model for the steady state $\left( 0, 0, 0.5, 0.5\right)$. Although in Fig. 2d the effect of $\lambda _2^\mathrm{r}$ on the stability of the steady state is not very clear, in Fig. 5a we can see that a decrease in $\lambda _2^\mathrm{r}$ leads to a shift of the graph below and a change in stability for very low $\lambda _2^\mathrm{r}$. Moreover, when we increase $q_\mathrm{r}$ (see $q_\mathrm{r}=1.1$ in Fig. 5b), the steady state becomes stable and we do not expect to have aggregation.

#### Remark 2

Note that $k=0$ is unstable for the trivial steady states [see Figs. 1 and 4a; a similar behaviour being observed in other non-local hyperbolic models (Eftimie et al. 2017)] and stable for the non-trivial states (see Figs. 2 and 4b; this behaviour is similar to the classical Turing-type instability).

## Numerical Results

To understand the behaviour of systems (1) and (12), we investigate them numerically. The aim of this section is to study the effect of the cell–cell interactions, baseline speed, proliferation and turning rates on the pattern formation for both models. The numerical results presented in this section are based on the investigation of the parameter sets that lead to pattern formation, i.e. predicted unstable wave numbers, as shown in the context of the linear stability analysis presented in the previous section. Note that similar patterns for different parameter sets or different initial conditions are not shown here for a better flow of this paper.

We use a time-splitting approach to discretise our model. We discretise the space–time plane choosing a time step $\Delta t=0.001$ and a space step $\Delta x=0.01$. We use a Crank–Nicolson scheme to propagate the solution of the diffusion terms for the parabolic equations (12), obtained with the formal parabolic limit of Eqs. (1). For the time propagation of the advection terms in both models (1) and (12), we use the Nessyahu–Tadmor scheme (Nessyahu and Tadmor 1990). Finally, for the time propagation of the reaction terms in (1) and (12), we use a fourth-order Runge–Kutta algorithm, where the integrals are further discretised using the Simpson’s rule. All simulations are performed on a domain of length $L=10$ with periodic boundary conditions (introduced to approximate the dynamics on an infinite domain). To deal with the integrals at the boundaries of the domain, we wrap them around the domain. The simulations ran for times up to $t = 2000$, but we show the dynamics for time that the patterns are more clear. The parameters used in the numerical simulations are listed in Table 2 in “Appendix A”.

### Pattern Formation for the Non-local Hyperbolic Model

Let us focus first on the numerical simulations for the non-local hyperbolic model (1). The initial conditions for the cancer cell populations are either small random perturbations of spatially homogeneous steady states

34 $$\begin{aligned} u_{i}^\pm (0, x)=u_{i}^{\pm , *}+\hbox {rand}(0,10^{-4}), \; i=1,2, \end{aligned}$$

or small random perturbations of rectangular-shaped aggregations located in the middle of the domain

35 $$\begin{aligned} u_{i}^\pm (0, x)=\left\{ \begin{array}{cc} 0.1+\hbox {rand}(0,10^{-4}), &{} \quad x\in (L/2-1,L/2+1),\\ 0, &{} \quad \text {everywhere}\; \text {else}. \end{array} \right. \end{aligned}$$

To begin we first run numerical simulations for small random perturbations of the steady states $\left( 0, 0, 0, 0\right)$ and $\left( 0, 0, 0.5, 0.5\right)$. As stated in the context of linear stability analysis in the previous section, the mutation rate, *M*, does not have any significant effect on the stability of the steady states. The numerical simulations obtained for $M=0.05$ show similar patterns (not shown here) with those for $M=0.0002$, but with $u_1$ population vanishing much faster and population $u_2$ reaching greater density values.

**Fig. 6:**
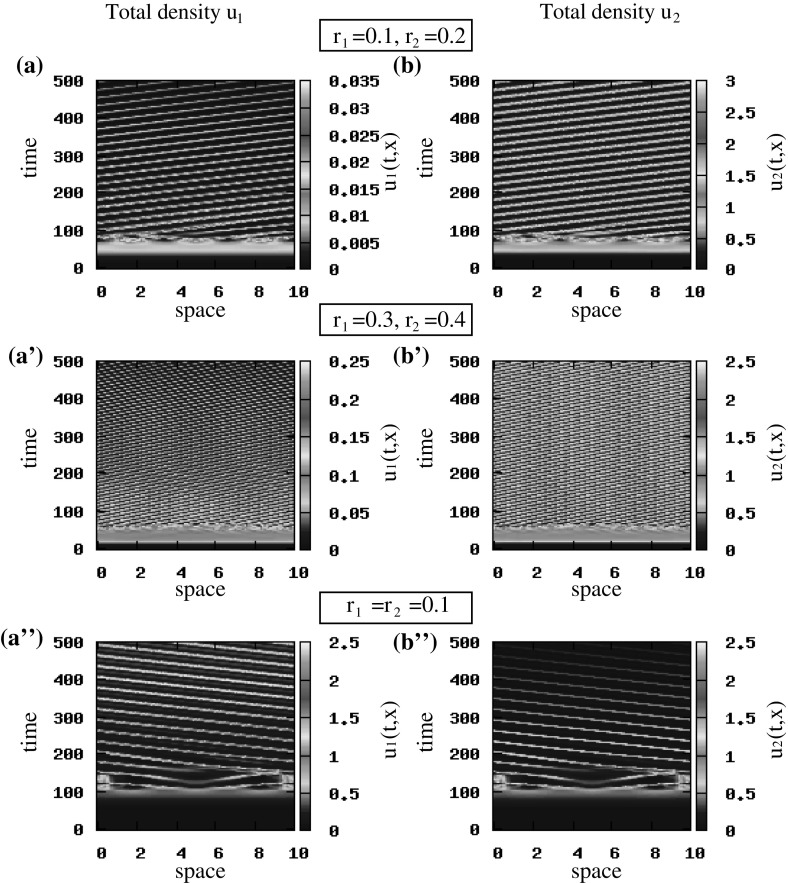
Patterns exhibited by the hyperbolic model (1). The initial conditions for the two cancer cell populations are described by small random perturbations of the steady state $\left( 0, 0, 0, 0\right)$ [see (34)]. **a**, **b** Total density of $u_1=u_1^++u_1^-$ and $u_2=u_2^++u_2^-$ for $r_1=0.1$ and $r_2=0.2$; **a’**, **b’** total density of $u_1=u_1^++u_1^-$ and $u_2=u_2^++u_2^-$ for $r_1=0.3$ and $r_2=0.4$; **a”**, **b”** total density of $u_1=u_1^++u_1^-$ and $u_2=u_2^++u_2^-$ for $r_1=r_2=0.1$. The rest of model parameters are given in Table 2 (Color figure online)

*The effect of proliferation rate on cancer cell movement and aggregation* As one of the key parameters of the stability of the steady state $\left( 0, 0, 0, 0\right)$ is the proliferation rate, in Fig. 6 we see that as the proliferation rates increase, e.g. $r_1=0.3$ and $r_2=0.4$ (see Fig. 6a’, b’) the $u_1$ and $u_2$ exhibit larger number of smaller rotating waves, as it was expected from the increased number of critical wave number shown in linear stability analysis (see Fig. 1c). Increasing further the proliferation rates $r_{1,2}$ (e.g. up to 0.7) leads to more aggregation waves. Choosing now the same proliferation rate for the two populations, e.g. $r_1=r_2=0.1$, we see in Fig. 6a”, b” an interesting effect of clonal competition, with population $u_1$ dominating the dynamics. This could be explained by the very low mutation rate and an equal competitive effect (i.e. $-r_{1}u_{1}u_{2}=-r_{2}u_{1}u_{2}$). Note that similar behaviours (where $u_{1}$ dominates the dynamics) have been observed for other sets of parameters with equal cell proliferation rates: e.g. $r_1=r_2=0.6$ or 0.7 (not shown here).

**Fig. 7:**
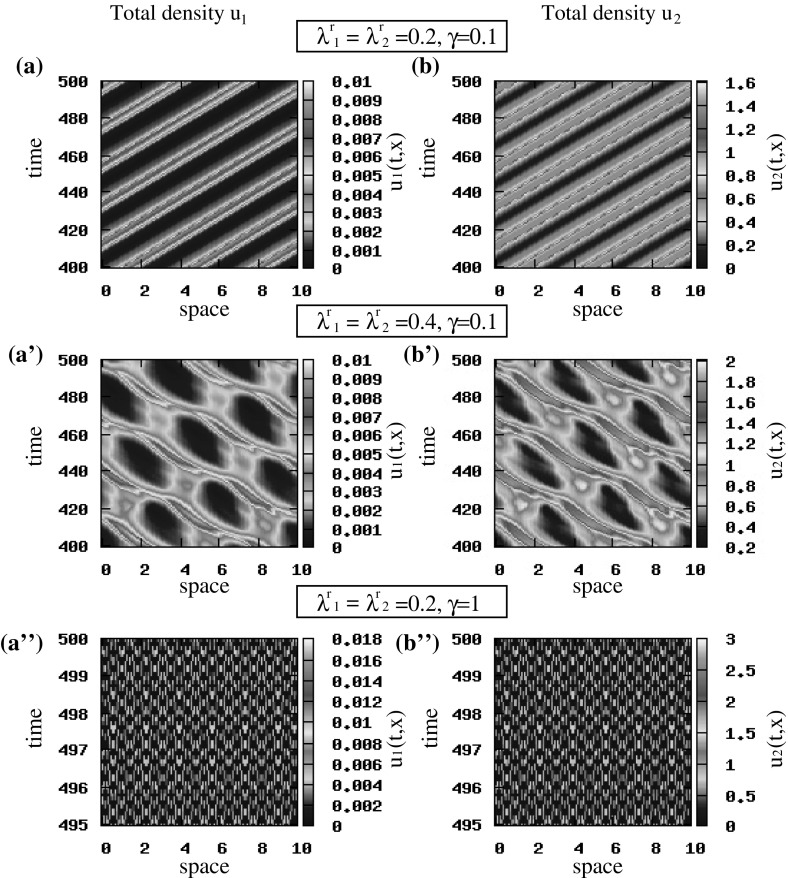
Patterns exhibited by the hyperbolic model (1). The initial conditions for the two cancer cell populations are described by small random perturbations of the steady state $\left( 0, 0, 0, 0\right)$ [see (34)]. **a**, **b** Total density of $u_1=u_1^++u_1^-$ and $u_2=u_2^++u_2^-$ for $\lambda ^\mathrm{r}_{1,2}=0.2$ and $\gamma =0.1$; **a’**, **b’** total density of $u_1=u_1^++u_1^-$ and $u_2=u_2^++u_2^-$ for $\lambda ^\mathrm{r}_{1,2}=0.4$ and $\gamma =0.1$; **a”**, **b”** total density of $u_1=u_1^++u_1^-$ and $u_2=u_2^++u_2^-$ for $\lambda ^\mathrm{r}_{1,2}=0.2$ and $\gamma =1$. The rest of model parameters are given in Table 2 (Color figure online)

*The effect of turning rates on cancer cell movement and aggregation* If we increase the baseline random turning rates, from $\lambda _i^\mathrm{r}=0.2$ to $\lambda _i^\mathrm{r}=0.4,\; i=1, 2$, we see in Fig. 7 that the cancer cell populations change their movement from rotating waves (panels a, b) to a combination of rotating waves and stationary pulses that are connected through splitting/merging behaviours that result from high random cell turning rates (panels a’, b’). However, if we reduce the baseline random turning rates, from $\lambda ^\mathrm{r}_{1,2}=0.2$ to $\lambda ^\mathrm{r}_{1,2}=0.1$, the rotating waves persist (not shown here). This behaviour was obtained for both steady states.

*The effect of baseline speed on cancer cell movement and aggregation* To investigate the effect of the baseline speed on pattern formation, we focus on the random initial conditions (34) and any of the two steady states (since perturbations of both states give rise to similar patterns). First we observe in Fig. 7a, b, a”, b” that an increase in cells’ speed (from $\gamma =0.1$ to $\gamma =1$) leads to a change in cells’ movement from rotating waves (panels a, b) to standing waves (panels a”, b” shown only for $495<t<500$ to improve the clarity of the figures). Moreover, we note that a reduction in the values of the baseline speed, $\gamma$, leads to the spread of populations over the whole domain. An example of such behaviour [for $\gamma =0.01$ and pulse-like initial conditions (35)] is shown in Fig. 10.

**Fig. 8:**
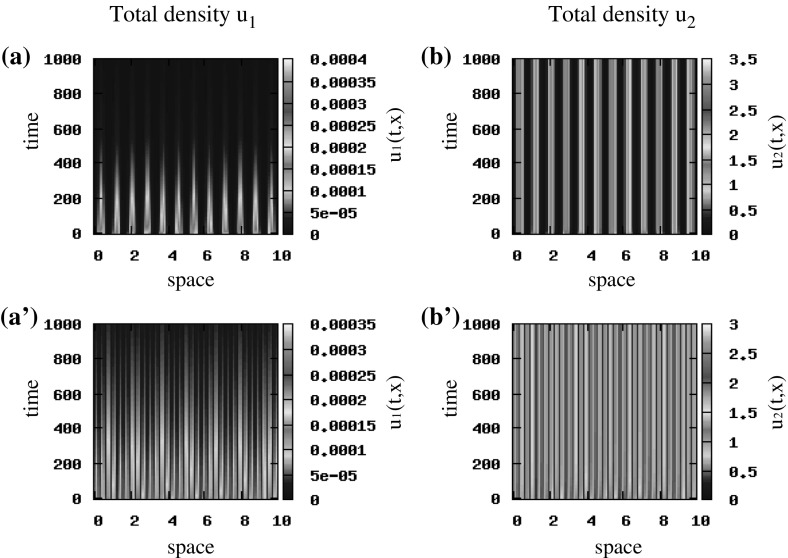
Patterns exhibited by the hyperbolic model (1). The initial conditions for the two cancer cell populations are described by small random perturbations of the steady state $\left( 0, 0, 0.5, 0.5\right)$ [see (34)]. **a**, **b** Total density of $u_1=u_1^++u_1^-$ and $u_2=u_2^++u_2^-$ for $q_\mathrm{a}=6, q_\mathrm{r}=0.1$ and $q_\mathrm{al}=3$; **a’**, **b’** total density of $u_1=u_1^++u_1^-$ and $u_2=u_2^++u_2^-$ for $q_\mathrm{a}=1.2, q_\mathrm{r}=6.5$ and $q_\mathrm{al}=3$. The rest of model parameters are given in Table 2 (Color figure online)

*The effect of attraction and repulsion on cancer cell movement and aggregation* Consider again the random initial conditions (34) applied to the steady state $(u_1^{+, *}, u_1^{-, *}$, $u_2^{+, *}, u_2^{-, *})=\left( 0, 0, 0.5, 0.5\right)$ [note that similar results have been obtained also for the steady state $\left( 0, 0, 0, 0\right)$—not shown here]. To investigate the effect of attraction and repulsion on pattern formation, we focus on two different cases: (i) $q_\mathrm{al}=3$ and $q_\mathrm{a}=6 \gg q_\mathrm{r}=0.1$ and (ii) $q_\mathrm{al}=3$ and $q_\mathrm{a}=1.2 \ll q_\mathrm{r}=6.5$. We see in Fig. 8a, b that the increase in cell–cell attraction leads to a (relatively) small number of large stationary cell aggregations. In contrast, the increase in cell–cell repulsion leads to the formation of a much larger number of smaller stationary cell aggregations (see Fig. 8a’, b’).

**Fig. 9:**
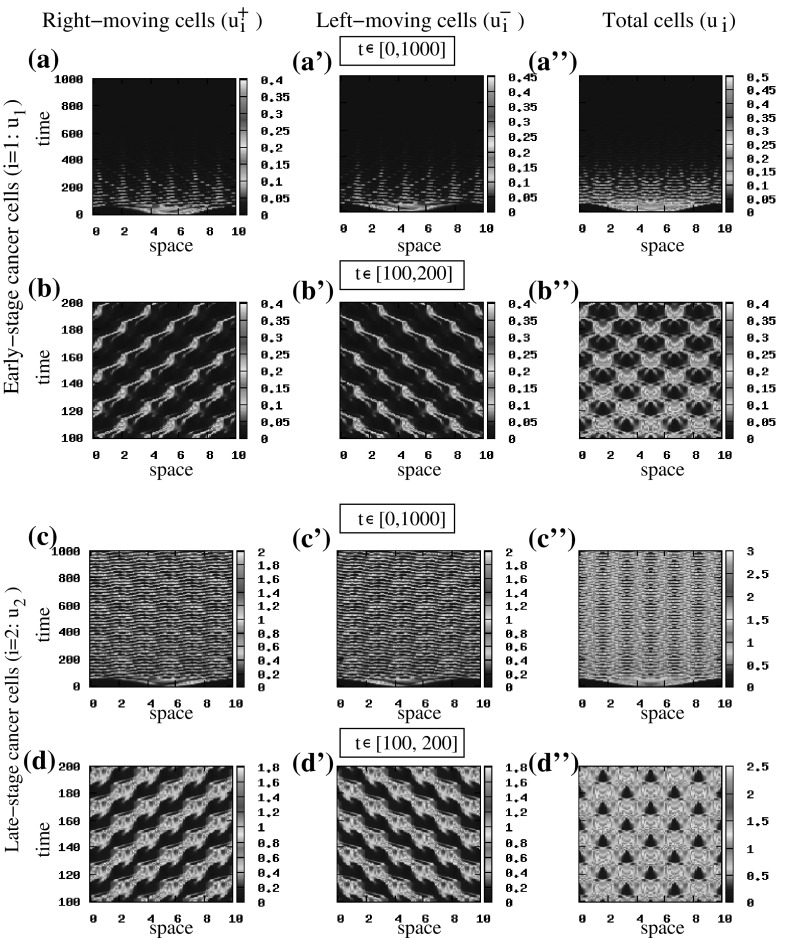
Patterns exhibited by the hyperbolic model (1) for $q_\mathrm{al}=0.5$ and $\lambda _{1,2}^\mathrm{r}=0.1$. The rest of model parameters as given in Table 2. The initial conditions for the two cancer cell populations consist of a rectangular pulse [see (35)]. **a**–**d** show the density of right-moving cancer cells $u_1^+$ (**a**, **b**) and $u_2^+$ (**c**, **d**). **a’**–**d’** show the density of left-moving cancer cells $u_1^-$ (**a’**, **b’**) and $u_2^-$ (**c’**, **d’**). **a”**–**d”** show the total density of cancer cells $u_1$ (**a”**, **b”**) and $u_2$ (**c”**, **d”**) (Color figure online)

*The effect of alignment on cancer cell movement and aggregation* Next we investigate the effect of alignment on collective cell movement and aggregation. In Fig. 9, we show the numerical results when $q_\mathrm{al}=0.5$ and $\lambda ^\mathrm{r}_{1,2}=0.1$, for initial conditions consisting of a rectangular pulse [see (35)]. The left-moving and right-moving cancer cell populations seem to exhibit a semi-zigzag pattern, characterised by a periodic transition between two different types of sub-patterns: stationary aggregations and travelling aggregations—see the first two columns in Fig. 9. This periodic transition seems to be similar to a heteroclinic connection. However, due to the complexity of the theory behind these heteroclinic connections in infinite-dimensional dynamical systems, it is beyond the purpose of this study to investigate them further. This will be the subject of future research.

We note here that increasing the magnitude of alignment to $q_\mathrm{al}=3$ leads again to rotating waves, while decreasing it to $q_\mathrm{al}=0$ leads to standing waves or stationary aggregations. In both cases, the patterns are similar to those shown in previous figures, and thus not included.

**Fig. 10:**
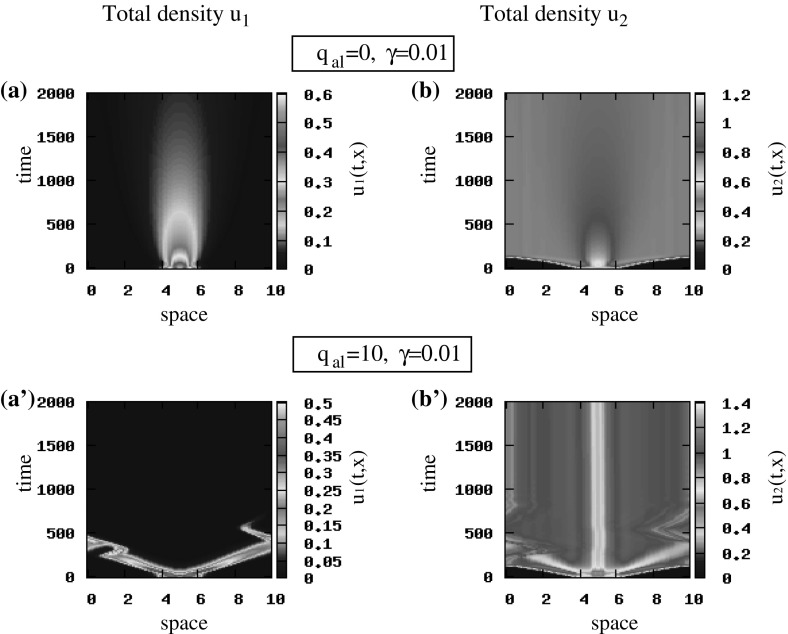
Patterns exhibited by the hyperbolic model (1) showing the cancer cell density for $\gamma =0.01$. The initial conditions for the two cancer cell populations consist of a rectangular pulse [see (35)]. **a**, **b** Total density of $u_1$ and $u_2$ for $q_\mathrm{al}=0$; **a’**, **b’** total density of $u_1$ and $u_2$ for $q_\mathrm{al}=10$. The rest of model parameters are given in Table 2 (Color figure online)

Finally, we investigate the combined effect of two parameters: cell alignment ($q_\mathrm{al}$) and cell baseline speed ($\gamma$). In contrast to the previous simulations, we now consider small speeds (i.e. $\gamma =0.01$) and slightly larger turning rates (i.e. $\lambda ^\mathrm{r}_{1,2}=0.2$). We see in Fig. 10a, b that when alignment is absent ($q_\mathrm{al}=0$), the $u_{1}$ cells form stationary aggregations (which eventually vanish due to the mutation term) and the $u_2$ cells spread throughout the whole domain.

When alignment is present ($q_\mathrm{al}>0$), we see in Fig. 10a’, b’ that some sub-populations of $u_1$ and $u_2$ cells move quickly to the left and to the right, reaching the domain boundaries. As in the previous case, the $u_{1}$ cells are eventually eliminated, while the $u_{2}$ cells spread throughout the whole domain. Note that similar results were obtained also with the random initial conditions (34)—not shown here.

#### Remark 3

To ensure that the cell aggregation patterns shown above did not depend on the boundary conditions, or on the numerical scheme used for discretisation, we also ran numerical simulations for the cases shown in Figs. 7a”, b” and 8a’, b’ when we doubled the domain size and refined the grid mesh. In all of these cases, the results showed no significant differences.

### Pattern Formation for the Limiting Parabolic Model

In this section, we run simulations for the limiting parabolic model given by (12). As in the hyperbolic model (1), we choose the initial conditions for the cancer cell populations to be small random perturbations of the spatially homogeneous steady states $\left( 0, 0\right)$ and $\left( 0, 1\right)$ (see Sect. 4.2)

36 $$\begin{aligned} u_{i}(0, x)=u_{i}^*+\hbox {rand}(0,10^{-4}), \; i=1,2, \end{aligned}$$

or small random perturbations of rectangular-shaped aggregations located in the middle of the domain

37 $$\begin{aligned} u_{i}(0, x)=\left\{ \begin{array}{cc} 0.2+\hbox {rand}(0,10^{-4}), &{} \quad x\in \left( {L}/{2}-1,{L}/{2}+1\right) \\ 0, &{} \quad \text {everywhere}\; \text {else}. \end{array} \right. \end{aligned}$$

**Fig. 11:**
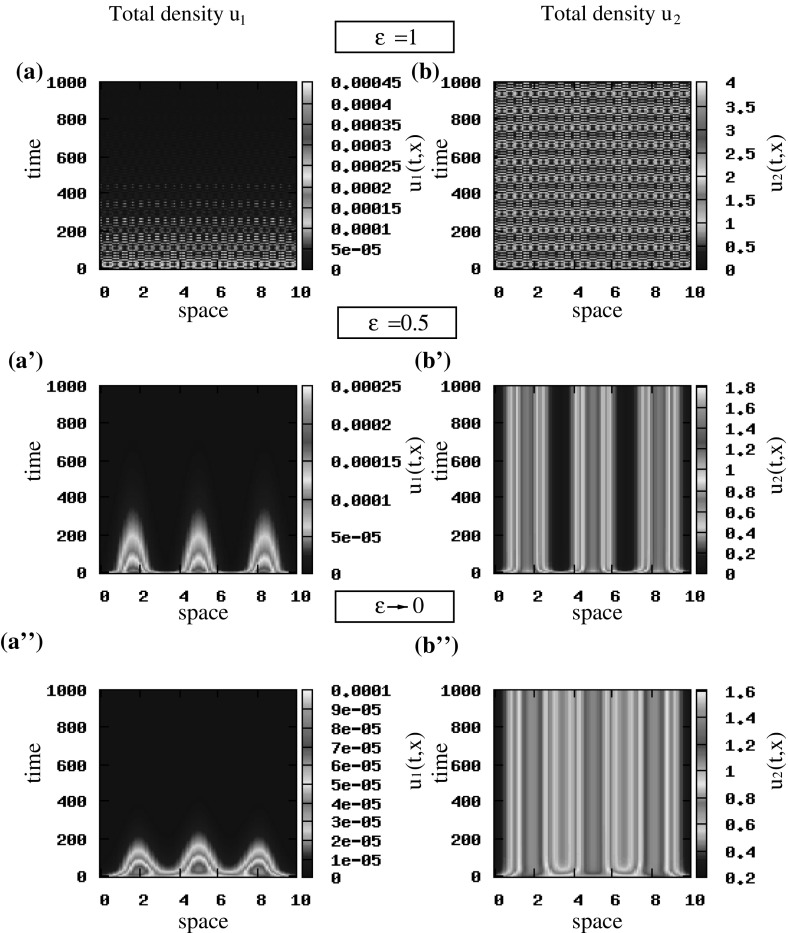
The spatiotemporal patterns obtained with the hyperbolic model (1) for $q_\mathrm{al}=0$ after scaling, and the parabolic model (12). **a**, **b** Standing waves obtained by (1) after scaling for $\epsilon =1$; **a’**, **b’** stationary pulses obtained by (1) after scaling for $\epsilon =0.5$; **a”**, **b”** stationary pulses obtained by (12) when $\epsilon \rightarrow 0$. The initial conditions for the two cancer cell populations are described by small random perturbation of the steady state $\left( 0, 0, 0.5, 0.5\right)$, for the rescaled hyperbolic model, and $\left( 0, 1\right)$, for the parabolic model [see (34) and (36)]. The rest of model parameters are given in Table 2 (Color figure online)

First, we assume that $q_\mathrm{al}=0$ and investigate how the travelling and stationary patterns predicted by the unstable wave numbers in the linear stability analysis of the hyperbolic model (1) (see Fig. 3), are preserved in the limit to the macroscopic parabolic model. If we consider small random perturbations of the hyperbolic steady state $\left( 0, 0, 0.5, 0.5\right)$, we see in Fig. 11a, b that for $\epsilon =1$ we obtain standing waves, and as $\epsilon$ decreases to 0.5, the cancer cells exhibit stationary pulses (Fig. 11a’, b’), as expected by the linear stability analysis results. Assume now $\epsilon \rightarrow 0$, and focus on the parabolic model (12). For initial conditions that are small random perturbations of the steady state $\left( 0, 1\right)$, we obtain stationary pulses (Fig. 11a”, b”) that are similar to the ones for the rescaled hyperbolic model when $\epsilon =0.5$ (see Fig. 11a’, b’). Similar pattern formation results are obtained for the hyperbolic steady state $\left( 0, 0, 0, 0\right)$ (and for the state $\left( 0, 0\right)$ corresponding to the parabolic model) for the case $\lambda ^\mathrm{r}_{1,2}=0.1$. If we increase the random turning rates to $\lambda ^\mathrm{r}_{1,2}=0.2$, we obtain stationary pulses for every $0<\epsilon \le 1$ in the rescaled hyperbolic model and for $\epsilon \rightarrow 0$ in the parabolic model, which was expected also from the linear stability analysis (see Fig. 3, where $Im\left( \lambda \left( k_j\right) \right) =0$ at the wave numbers where $Re\left( \lambda \left( k_j\right) \right) >0$).

**Fig. 12:**
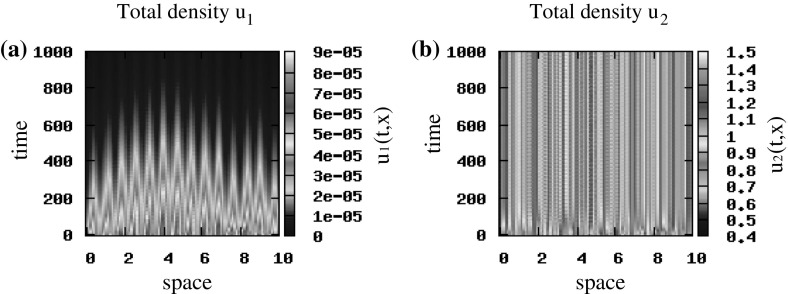
Patterns exhibited by the parabolic model (12). The initial conditions for the two cancer cell populations are described by small random perturbations of the steady state $\left( 0, 1\right)$ [see (36)]. Total density of $u_1$ and $u_2$ for $q_\mathrm{a}=6, q_\mathrm{r}=0.1$ and $q_\mathrm{al}=0$. The rest of the model parameters are given in Table 2 (Color figure online)

**Fig. 13:**
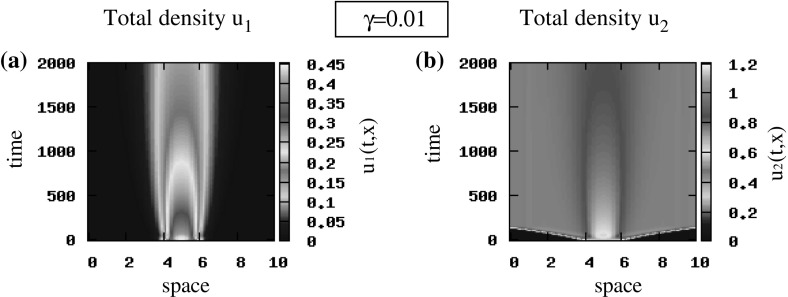
Patterns exhibited by the parabolic model (12) showing the cancer cell density for $\gamma =0.01$. The initial conditions for the two cancer cell populations consist of a rectangular pulse [see (37)]. **a**, **b** Total density of $u_1$ and $u_2$ for $q_\mathrm{a}=1.2, q_\mathrm{r}=0.1$ and $q_\mathrm{al}=0$. The rest of model parameters are given in Table 2 (Color figure online)

Finally, since we expect that the repulsive–attractive interactions could affect also the dynamics of the parabolic model, in Fig. 12 we investigate the effect of increasing the attraction strength to $q_{a}=6$ (same as in Fig. 8a, b, but here $q_\mathrm{al}=0$). We see that the parabolic model exhibits a large number of small stationary pulses, similar to those obtained by the hyperbolic model in Fig. 8.

Reducing now the speed (and assuming pulse-like initial conditions (37), as for the hyperbolic model), we notice that the patterns displayed in Fig. 13a, b are similar to those obtained for the hyperbolic model presented in Fig. 10a, b.

From the numerical simulations of both models, we conclude that the hyperbolic model can exhibit both moving and stationary behaviours, in contrast to the parabolic model that can exhibit only stationary behaviours.

## Conclusion and Discussion

In this paper, we introduced a one-dimensional non-local hyperbolic model describing the interactions between heterogeneous cancer cells. We developed a model where non-local turning rates are included and incorporate all three social interactions: attraction, repulsion and alignment, that play a crucial role in cell movement and aggregation. We assumed that a cell changes its movement direction only after weighing the information received from left and right, speeding up and slowing down to catch up with the surrounding cells, or to avoid collisions. The mutation terms and the proliferation terms are chosen to take into account the movement of cells in opposite directions. We should emphasise that we assumed that cancer cells can detect cells that are in front and behind them.

Then, we reduced our non-local hyperbolic model to a non-local parabolic model for cellular aggregations, since parabolic models have been commonly used in the study of the formation and movement of cell and animal aggregations. To this end, we considered a formal parabolic limit which assumed very large turning rates and very large speeds.

Linear stability analysis of both hyperbolic and parabolic model was used to examine the possibility of cell aggregations to form (as a result of spatial perturbations of the steady states). The results showed that for the hyperbolic model, the dispersion relation had nonzero imaginary part (hence it was possible to have Hopf bifurcations, in addition to real bifurcations). In contrast, the dispersion relation for the parabolic model had zero imaginary part (hence aggregations could arise only via real bifurcations). Finally, we ran simulations for the hyperbolic and the related parabolic model and compared the results: the hyperbolic model was more rich in patterns, showing moving cell aggregations, while the parabolic model exhibited mainly stationary cell aggregations. These numerical results are consistent with the linear results obtained via stability analysis, emphasising the more complex behaviour of the hyperbolic model.

The model presented in this paper focussed mainly on the interactions between cells, excluding the important role of ECM on cellular adhesion, movement and aggregation. A straightforward future research direction is to include ECM density in this non-local model that incorporates cell alignment.

## Appendix A: Non-dimensionalisation and Parameter Values

### Non-dimensionalisation of the Model

To obtain the non-dimensional system (1), we use the following quantities:

38 $$\begin{aligned} \tilde{t}=\dfrac{t}{\tau }, \quad \tilde{x}=\dfrac{x}{L_0}, \quad \tilde{u}_i^\pm =\dfrac{u_i^\pm }{k_u}, \quad \tilde{u}_i=\dfrac{u_i}{k_u}, \quad i=1, 2, \quad \tilde{R_\mathrm{s}}=\dfrac{R_\mathrm{s}}{L_{0}},\quad \tilde{s}=\dfrac{s}{L_{0}}. \end{aligned}$$

The length scale, $L_{0}$, is in the range of 0.1–1 cm and is defined as the maximum invasion distance of the cancer cells at the early stage of invasion (Anderson et al. 2000). The time scale is defined as $\tau :=L_{0}^2/D_{\tau }$, where $D_{\tau }$ is a reference chemical diffusion coefficient, e.g. $\sim 10^{-6}\hbox {cm}^2\hbox { s}^{-1}$ (Bray 2001). Furthermore, we rescale the cancer cells with $k_u$. Here, $k_u$ is the carrying capacity of the cancer cell populations and it is taken to be $\sim 6.7\cdot 10^7\hbox {cell volume}^{-1}$ (Domschke et al. 2014). For the kernels $K_j\left( s\right) , j=a, r, al$, we define (as in Domschke et al. 2014) the dimensionless functions $\tilde{K_j}\left( \tilde{s}\right) , i=1,2,$ given by

$$\begin{aligned} \tilde{K_j}\left( \tilde{s}\right) :=L_{0}K_j\left( L_0\tilde{s}\right) =L_{0}K_j\left( s\right) , j=a, r, al. \end{aligned}$$

The non-dimensionalisation of the model is straightforward, and we obtain the following dimensionless parameters:

39 $$\begin{aligned} \tilde{q_j}= & {} \dfrac{k_u}{L_0}q_j,\; j=a, r, al,\; \tilde{\gamma }=\dfrac{\tau }{L_0}\gamma ,\; \tilde{\lambda }_i^\mathrm{r}=\tau \lambda _i^\mathrm{r},\;\nonumber \\ \tilde{\lambda }_i^{\mathrm{b}}= & {} \tau \lambda _i^{\mathrm{b}},\; \tilde{M}=\tau M, \; \tilde{r_i}=\tau r_i,\;\; i=1, 2. \end{aligned}$$

After dropping the tildes for notation convenience, we obtain the non-dimensionalised hyperbolic model (1).

Note that we assume that both populations $u_1$ and $u_2$ proliferate in a logistic manner to describe the observed slow down in tumour growth following the loss of nutrients (Laird 1964). The growth functions $G_1$ and $G_2$ for populations $u_1$ and $u_2$, respectively, are then given by the following relations

40 $$\begin{aligned} G_i\left( \underline{u}\right) =r_i\dfrac{u_i}{2}\left( 1-\dfrac{u_1+u_2}{k_u}\right) , \;\; i=1, 2, \end{aligned}$$

which after the non-dimensionalisation lead to the logistic growth functions with unit-valued carrying capacity for the cells that appear in model (1).

### A.2 Summary of Model Variables and Parameters

We present here two tables with the model variables and parameters. In Table 1, we list the model variables with their units. In Table 2, we list the parameters of our model and their corresponding units and non-dimensional values used in the simulations.

**Table 1:** A list of model variables with their units

Variable	Description	Dimensional units
$u_1^+$	Right-moving early-stage cancer cell density	cell/length
$u_1^-$	Left-moving early-stage cancer cell density	cell/length
$u_1$	Total early-stage cancer cell density	cell/length
$u_2^+$	Right-moving late-stage cancer cell density	cell/length
$u_2^-$	Left-moving late-stage cancer cell density	cell/length
$u_2$	Total late-stage cancer cell density	cell/length

Since we are in 1D, length and volume coincide and we express the variables in terms of domain length

*Parameter estimation*

- The sensing radius was based on the range of values given in Armstrong et al. (2006) and Gerisch and Chaplain (2008). In this study, we choose $\tilde{R_\mathrm{s}}=1$.
- Attraction, repulsion and alignment ranges, $s_j, \; j=a, r, al$, were chosen to be smaller or equal to sensing radius, with $s_\mathrm{r}<s_\mathrm{al}<s_\mathrm{a}$ (Green et al. 2010).
- Experimental studies (Cillo et al. 1987; Hill et al. 1984; Mareel et al. 1991) have shown that the mutation rate ranges between $10^{-8}$/ cell/generation and $10^{-5}$/ cell/generation. We assume an average growing cell population of $\approx 10^4$ cells/generation, a 1-day generation of cells (since the doubling time is about 1.2 days) (den Breems and Eftimie 2016). Thus, the mutation rate will range between $M=10^{-4}$/day and $M=0.1$/day, which for $\tau =10^5$ s corresponds to a non-dimensional value of the mutation rate ranging between $\tilde{M}=0.000116$ and $\tilde{M}=0.116$ (for highly aggressive tumours). In this study, we choose $\tilde{M}=0.0002$.
- Various experimental studies (Cunningham and You 2015; Morani et al. 2014) have shown that doubling times for tumour cells range from 1 to 10 days. This corresponds to growth rates between $\ln \left( 2\right) /10=0.07 \;\text{(days) }^{-1}$ and $\ln \left( 2\right) /1 =0.7\; \text{(days) }^{-1}$. We choose $\tau =10^5$ s; thus, $\tilde{r_i}\in \left( 0.081, 0.81\right) , \; i=1, 2$. In this study, we assume that $\tilde{r_1}=0.1$ and $\tilde{r_2}=0.2$.

**Table 2:** A list of model parameters with their units and their non-dimensional values, which we used during numerical simulations

Param.	Description	Dimensional units	Non-dim. value ($\tilde{p}$)	Reference
$R_\mathrm{s}$	Sensing radius	length	1	Armstrong et al. (2006)
$\gamma$	Constant baseline speed of cancer cells	length/time	0.1	Estimated
$q_\mathrm{a}$	Magnitude of attraction	$\hbox {length}^2/\hbox {cell}$	1.2	Estimated
$q_\mathrm{r}$	Magnitude of repulsion	$\hbox {length}^2/\hbox {cell}$	0.1	Estimated
$q_\mathrm{al}$	Magnitude of alignment	$\hbox {length}^2/\hbox {cell}$	3	Estimated
$s_\mathrm{a}$	Attraction range	length	1	Estimated
$s_\mathrm{r}$	Repulsion range	length	0.25	Estimated
$s_\mathrm{al}$	Alignment range	length	0.5	Estimated
$m_\mathrm{a}$	Width of attraction kernel	length	1/8	Estimated
$m_\mathrm{r}$	Width of repulsion kernel	length	0.25/8	Estimated
$m_\mathrm{al}$	Width of alignment kernel	length	0.5/8	Estimated
*M*	Mutation rate	1/time	0.0002	Calabrese and Shibata (2010) and Mareel et al. (1991)
$\lambda _1^\mathrm{r}$	Baseline random turning rate of cancer cell population $u_1$	1/time	0.2	Estimated
$\lambda _2^\mathrm{r}$	Baseline random turning rate of cancer cell population $u_2$	1/time	0.2	Estimated
$\lambda _1^\mathrm{b}$	Biased turning rate of cancer cell population $u_1$	1/time	0.8	Estimated
$\lambda _2^\mathrm{b}$	Biased turning rate of cancer cell population $u_2$	1/time	1	Estimated
$r_1$	Growth rate of $u_1$	1/time	0.1	Morani et al. (2014)
$r_2$	Growth rate of $u_2$	1/time	0.2	Morani et al. (2014)

## Appendix B: Derivation of the Parabolic Model

Here, we present in detail the derivation of the parabolic model (12). The technique used to reduce our non-local hyperbolic model to a non-local parabolic model has been described previously in various other papers (see, for instance, Hillen and Levine 2003; Hillen and Stevens 2000).

As mentioned in Sect. 3, we add and subtract Eqs. (1a)–(1b) for population $u_1$, and similarly Eqs. (1c)–(1d) for population $u_2$, to obtain the following system:

41a $$\begin{aligned} \dfrac{\partial u_1}{\partial t}+\gamma \dfrac{\partial \upsilon _1}{\partial x}+\gamma \dfrac{\partial }{\partial x}\left( u_1g\left[ \underline{u}\right] \right)&=-Mu_1+R_1\left( \underline{u}\right) , \end{aligned}$$

41b $$\begin{aligned} \dfrac{\partial \upsilon _1}{\partial t}+\gamma \dfrac{\partial u_1}{\partial x}+\gamma \dfrac{\partial }{\partial x}\left( \upsilon _1g\left[ \underline{u}\right] \right)&=f_1\left[ \underline{u}\right] u_1-h_1\left[ \underline{u}\right] \upsilon _1-M\upsilon _1, \end{aligned}$$

41c $$\begin{aligned} \dfrac{\partial u_2}{\partial t}+\gamma \dfrac{\partial \upsilon _2}{\partial x}+\gamma \dfrac{\partial }{\partial x}\left( u_2g\left[ \underline{u}\right] \right)&=Mu_1+R_2\left( \underline{u}\right) , \end{aligned}$$

41d $$\begin{aligned} \dfrac{\partial \upsilon _2}{\partial t}+\gamma \dfrac{\partial u_2}{\partial x}+\gamma \dfrac{\partial }{\partial x}\left( \upsilon _2g\left[ \underline{u}\right] \right)&=f_2\left[ \underline{u}\right] u_2-h_2\left[ \underline{u}\right] \upsilon _2, \end{aligned}$$

where

42 $$\begin{aligned} R_i\left( \underline{u}\right)&=r_iu_i\left( 1-u_1-u_2\right) , \end{aligned}$$

43 $$\begin{aligned} f_i\left[ \underline{u}\right]&=\lambda _{u_i}^-\left[ u_1^+, u_1^-, u_2^+, u_2^-\right] -\lambda _{u_i}^+\left[ u_1^+, u_1^-, u_2^+, u_2^-\right] , \end{aligned}$$

44 $$\begin{aligned} h_i\left[ \underline{u}\right]&=\lambda _{u_i}^-\left[ u_1^+, u_1^-, u_2^+, u_2^-\right] +\lambda _{u_i}^+\left[ u_1^+, u_1^-, u_2^+, u_2^-\right] , \;\;\;i=1, 2. \end{aligned}$$

Next, we differentiate Eqs. (41a) and (41c) with respect to *t*, and Eqs. (41b) and (41d) with respect to *x*, to obtain:

45 $$\begin{aligned} \dfrac{\partial ^2 u_1}{\partial t^2}+\gamma \dfrac{\partial ^2 \upsilon _1}{\partial t\partial x}+\gamma \dfrac{\partial ^2 }{\partial t\partial x}\left( u_1g\left[ \underline{u}\right] \right)&=-M\dfrac{\partial u_1}{\partial t}+\dfrac{\partial }{\partial t}R_1\left( \underline{u}\right) ,\end{aligned}$$

46 $$\begin{aligned} \dfrac{\partial ^2\upsilon _1}{\partial x\partial t}+\gamma \dfrac{\partial ^2 u_1}{\partial x^2}+\gamma \dfrac{\partial ^2 }{\partial x^2}\left( \upsilon _1g\left[ \underline{u}\right] \right)&=\dfrac{\partial }{\partial x}\left( u_1f_1\left[ \underline{u}\right] \right) -\upsilon _1\dfrac{\partial }{\partial x}h_1\left[ \underline{u}\right] \nonumber \\&\quad -\left( h_1\left[ \underline{u}\right] + M\right) \dfrac{\partial \upsilon _1}{\partial x}, \end{aligned}$$

47 $$\begin{aligned} \dfrac{\partial ^2 u_2}{\partial t^2}+\gamma \dfrac{\partial ^2 \upsilon _2}{\partial t\partial x}+\gamma \dfrac{\partial ^2 }{\partial t\partial x}\left( u_2g\left[ \underline{u}\right] \right)&=M\dfrac{\partial u_1}{\partial t}+\dfrac{\partial }{\partial t}R_2\left( \underline{u}\right) , \end{aligned}$$

48 $$\begin{aligned} \dfrac{\partial ^2 \upsilon _2}{\partial x\partial t}+\gamma \dfrac{\partial ^2 u_2}{\partial x^2}+\gamma \dfrac{\partial ^2 }{\partial x^2}\left( \upsilon _2g\left[ \underline{u}\right] \right)&=\dfrac{\partial }{\partial x}\left( u_2f_2\left[ \underline{u}\right] \right) -\upsilon _2\dfrac{\partial }{\partial x}h_2\left[ \underline{u}\right] \nonumber \\&\quad -h_2\left[ \underline{u}\right] \dfrac{\partial \upsilon _2}{\partial x}. \end{aligned}$$

We eliminate the terms $\frac{\partial ^2\upsilon _1}{\partial t\partial x}$ and $\frac{\partial ^2\upsilon _2}{\partial t\partial x}$ from Eqs. (45)–(46) and (47)–(48), respectively. Moreover, we assume that the flows $\upsilon _i, i=1, 2$, are zero at the boundaries. Using Eqs. (41a) and (41c), we replace $\upsilon _i, \;i=1, 2$, with

49 $$\begin{aligned} \upsilon _i=\int ^x\left( -\dfrac{1}{\gamma }\dfrac{\partial u_i}{\partial t}-\dfrac{\partial }{\partial x}\left( u_ig\left[ \underline{u}\right] \right) +\left( -1\right) ^i\dfrac{M}{\gamma }u_1+\dfrac{1}{\gamma }R_i\left( \underline{u}\right) \right) \mathrm {d}s, \end{aligned}$$

and $$\dfrac{\partial \upsilon _i}{\partial x}, i=1, 2$$, with

50 $$\begin{aligned} \dfrac{\partial \upsilon _i}{\partial x}=-\dfrac{1}{\gamma }\dfrac{\partial u_i}{\partial t}-\dfrac{\partial }{\partial x}\left( u_ig\left[ \underline{u}\right] \right) +\left( -1\right) ^i\dfrac{M}{\gamma }u_1+\dfrac{1}{\gamma }R_i\left( \underline{u}\right) . \end{aligned}$$

Therefore, we obtain the following second-order equations

51 $$\begin{aligned}&\dfrac{\partial ^2 u_1}{\partial t^2}-\gamma ^2\dfrac{\partial ^2 u_1}{\partial x^2}+\gamma \dfrac{\partial }{\partial x}\left( u_1f_1\left[ \underline{u}\right] \right) +\gamma \dfrac{\partial ^2}{\partial t\partial x}\left( u_1 g\left[ \underline{u}\right] \right) +M\dfrac{\partial u_1}{\partial t}-\dfrac{\partial }{\partial t}\left( R_1\left( \underline{u}\right) \right) \nonumber \\&\quad +\left( -\gamma \dfrac{\partial ^2}{\partial x^2}g\left[ \underline{u}\right] -\dfrac{\partial }{\partial x}h_1\left[ \underline{u}\right] \right) \bigg (\int ^x\bigg (-\dfrac{\partial u_1}{\partial t}-\gamma \dfrac{\partial }{\partial x}\left( u_1g\left[ \underline{u}\right] \right) \nonumber \\&\quad -Mu_1+R_1\left( \underline{u}\right) \bigg )\mathrm {d}s\bigg )\nonumber \\&\quad +\left( -2\gamma \dfrac{\partial }{\partial x}g\left[ \underline{u}\right] -h_1\left[ \underline{u}\right] -M\right) \left( -\dfrac{\partial u_1}{\partial t}-\gamma \dfrac{\partial }{\partial x}\left( u_1g\left[ \underline{u}\right] \right) -Mu_1+R_1\left( \underline{u}\right) \right) \nonumber \\&\quad -\gamma g\left[ \underline{u}\right] \dfrac{\partial }{\partial x}\left( -\dfrac{\partial u_1}{\partial t}-\gamma \dfrac{\partial }{\partial x}\left( u_1g\left[ \underline{u}\right] \right) -Mu_1+R_1\left( \underline{u}\right) \right) =0, \end{aligned}$$

52 $$\begin{aligned} \nonumber \\&\dfrac{\partial ^2 u_2}{\partial t^2}-\gamma ^2\dfrac{\partial ^2 u_2}{\partial x^2}+\gamma \dfrac{\partial }{\partial x}\left( u_2f_2\left[ \underline{u}\right] \right) +\gamma \dfrac{\partial ^2}{\partial t\partial x}\left( u_2 g\left[ \underline{u}\right] \right) -M\dfrac{\partial u_1}{\partial t}-\dfrac{\partial }{\partial t}\left( R_2\left( \underline{u}\right) \right) \nonumber \\&\quad +\left( -\gamma \dfrac{\partial ^2}{\partial x^2}g\left[ \underline{u}\right] -\dfrac{\partial }{\partial x}h_2\left[ \underline{u}\right] \right) \bigg (\int ^x\bigg (-\dfrac{\partial u_2}{\partial t}-\gamma \dfrac{\partial }{\partial x}\left( u_2g\left[ \underline{u}\right] \right) \nonumber \\&\quad +Mu_1+R_2\left( \underline{u}\right) \bigg )\mathrm {d}s\bigg )\nonumber \\&\quad +\left( -2\gamma \dfrac{\partial }{\partial x}g\left[ \underline{u}\right] -h_2\left[ \underline{u}\right] \right) \left( -\dfrac{\partial u_2}{\partial t}-\gamma \dfrac{\partial }{\partial x}\left( u_2g\left[ \underline{u}\right] \right) +Mu_1+R_2\left( \underline{u}\right) \right) \nonumber \\&\quad -\gamma g\left[ \underline{u}\right] \dfrac{\partial }{\partial x}\left( -\dfrac{\partial u_2}{\partial t}-\gamma \dfrac{\partial }{\partial x}\left( u_2g\left[ \underline{u}\right] \right) +Mu_1+R_2\left( \underline{u}\right) \right) =0. \end{aligned}$$

We introduce now a small dimensionless parameter $\epsilon > 0$ and consider the following parameter scaling (see Sect. 3):

(i) $$\lambda ^\mathrm{r}_i=\dfrac{\bar{\lambda }_i^{\mathrm{r}}}{\epsilon ^2},\;\;\; \lambda ^\mathrm{b}_i=\dfrac{\bar{\lambda }_i^{\mathrm{b}}}{\epsilon ^2},\; i=1, 2$$,
(ii) $$\gamma =\dfrac{\bar{\gamma }}{\epsilon }$$,
(iii) $$g\left[ \underline{u}\right] =\epsilon \bar{g}\left[ \underline{u}\right] $$,
(iv) $$p\left( y^\pm \left[ \underline{u}\right] \right) =\epsilon \bar{p}\left( y^\pm \left[ \underline{u}\right] \right) $$,

and the rescaled functionals $f_i\left[ \underline{u}\right]$ and $h_i\left[ \underline{u}\right]$ described in relations (10) and (11), respectively.

Substituting these rescaled parameters and functions into Eqs. (51)–(52) and multiplying with $\epsilon ^2$, taking the limit as $\epsilon \rightarrow 0$ and dropping the bars (for simplicity), leads to the following parabolic equations

53a $$\begin{aligned} \dfrac{\partial u_1}{\partial t}&= D_{u_1}\dfrac{\partial ^2 u_1}{\partial x^2}-\dfrac{\gamma \lambda _1^\mathrm{b}}{2\lambda _1^\mathrm{r}}\dfrac{\partial }{\partial x}\left( u_1f\left[ \underline{u}\right] \right) -\gamma \dfrac{\partial }{\partial x}\left( u_1g\left[ \underline{u}\right] \right) \nonumber \\&\quad -Mu_1+R_1\left( \underline{u}\right) , \end{aligned}$$

53b $$\begin{aligned} \dfrac{\partial u_2}{\partial t}&= D_{u_2}\dfrac{\partial ^2 u_2}{\partial x^2}-\dfrac{\gamma \lambda _2^\mathrm{b}}{2\lambda _2^\mathrm{r}}\dfrac{\partial }{\partial x}\left( u_2f\left[ \underline{u}\right] \right) -\gamma \dfrac{\partial }{\partial x}\left( u_2g\left[ \underline{u}\right] \right) \nonumber \\&\quad +Mu_1+R_2\left( \underline{u}\right) , \end{aligned}$$

where $D_{u_i}=\dfrac{\left( \gamma \right) ^2}{2\lambda ^\mathrm{r}_i},\; i=1, 2$, are the diffusion coefficients.
